# Supplementary material for: In Silico Tools to Extract the Drug Design Information Content of Degradation Data: The Case of PROTACs Targeting the Androgen Receptor
Source: Molecules. 2023 Jan 26;28(3):1206. doi: 10.3390/molecules28031206 (PMC9919651; doi:10.3390/molecules28031206)
Supplement: Supplementary file 1 [file molecules-28-01206-s001.zip › molecules-2130351-supplementary.pdf]

Supporting Information

In silico tools to extract the drug design information content of degradation data: the case of PROTACs targeting the Androgen Receptor

Giulia Apprato, Giulia D'Agostini, Paolo Rossetti, Giuseppe Ermondi and Giulia Caron *

Molecular Biotechnology and Health Sciences Department, University of Torino, Via Quarello, 15, 10135 Torino, Italy

* Correspondence: gulia.caron@unito.it

Supplementary Figures

Figure S1: Dataset distribution in CRBN, VHL-recruiting degraders

Figure S2: ARV-110 Murcko scaffold and skeleton

Figure S3: Similarity chart

Figure S4: Degradation cliff schematic procedure

Figure S5: E3 ligand-related Degradation Cliff pairs

Figure S6: Linker-degradation relationship structures

Figure S7: c71-c77 ternary complex comparison (3D view)

Figure S8: c48-c52 ternary complex comparison (ligand interaction and table of interactions)

Figure S9: c48-c52 ternary complex comparison (3D view)

Figure S10: VHL, CRBN molecular descriptors distribution

Figure S11: Correlation plot reported for VHL, CRBN, and the entire dataset

Supplementary Tables

Table S1: PROTACs SMILES and 2D structure

Table S2: PROTACs activity and active/inactive classification

Table S3: Matched Molecular Pair clusters obtained for the entire degrader

Table S4: Matched Molecular Pair clusters obtained for the warhead

Table S5: Matched Molecular Pair clusters obtained for the E3 ligand

Table S6: Matched Molecular Pair clusters obtained for the linker

Table S7: Degradation Cliff couples

Table S8: performance metrics of 0.1uM activity VHL classification models

Table S9: performance metrics of 0.1uM activity CRBN classification models

Table S10: performance metrics of 0.1uM activity entire dataset classification models

Table S11: performance metrics of 1uM activity VHL classification models

Table S12: performance metrics of 1uM activity CRBN classification models

Table S13: performance metrics of 1uM activity entire dataset classification models

Table S14: y-randomization reported for each model: VHL, CRBN, and the entire dataset

Table S15: performance metrics of 3-descriptors model (0.1uM)

Table S16: Crystallographic structures employed in ternary complex modeling

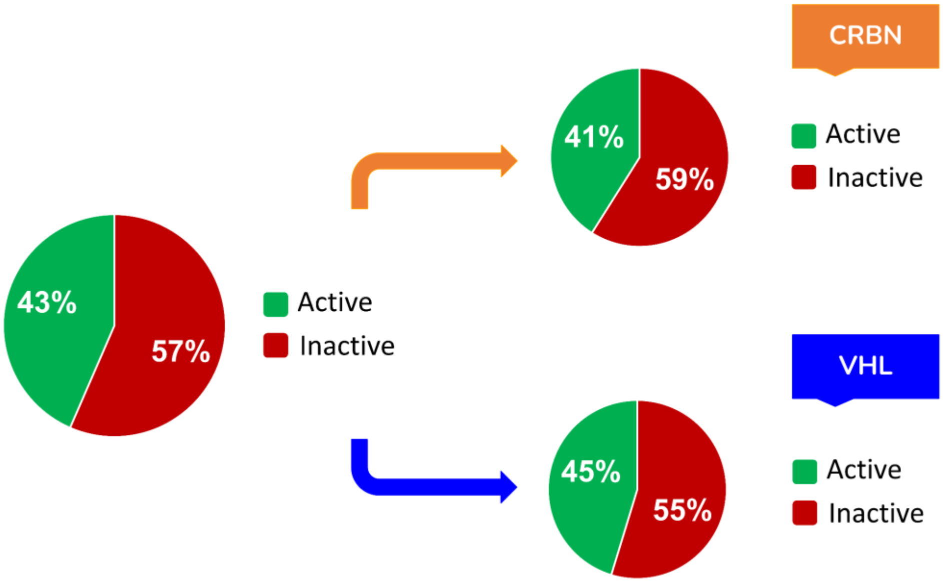

Figure S1. Representation of the distribution of the entire dataset at 0.1uM concentration activity (pie chart on the left) and distribution of CRBN and VHL set activity at the same concentration.

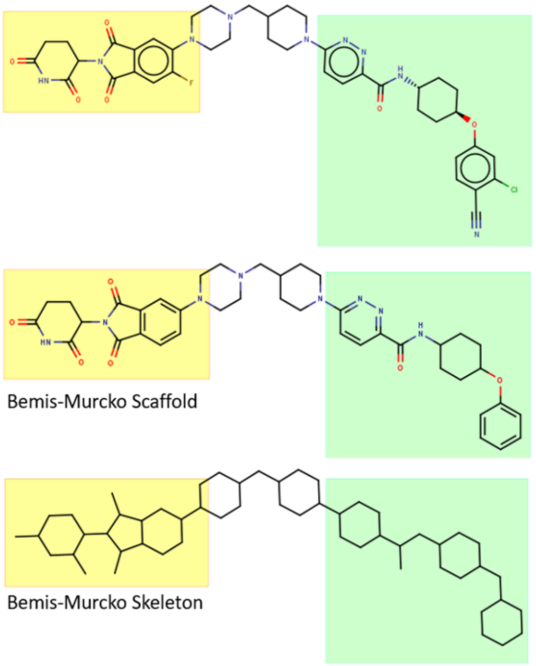

Figure S2. The Bemis Murcko framework analysis: Murcko skeleton and scaffold of ARV-110.

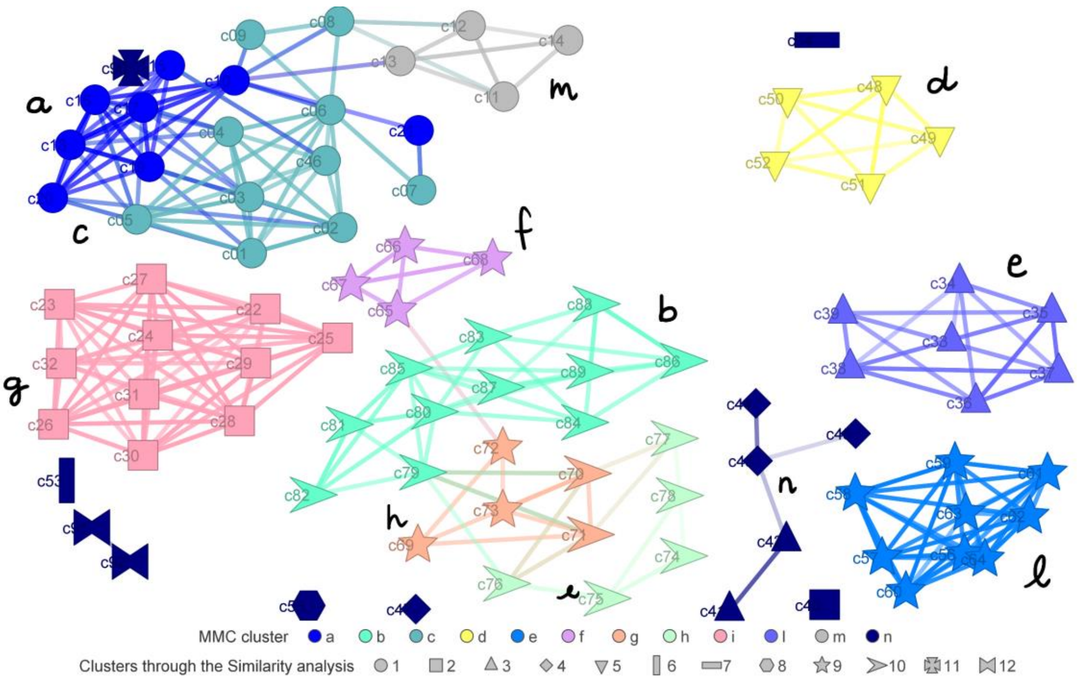

Figure S3. In the Similarity chart each compound is placed in the X-Y plane considering similar neighborhood relations; similar degraders are clustered together and connected by a line. Degraders are colored according to the Matched Molecular Pair clusters identified (a-n) and are shaped according to the similarity analysis identified clusters (similarity set to 90%).

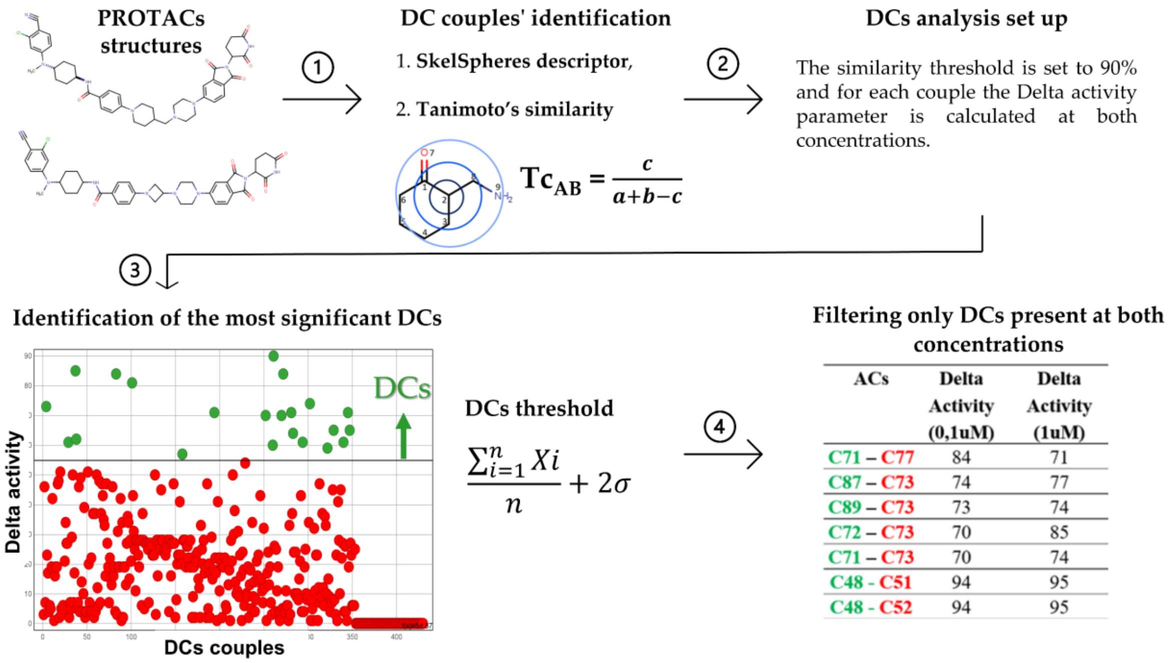

Figure S4. The schematic procedure adopted to identify the most significant Degradation Cliffs is reported.

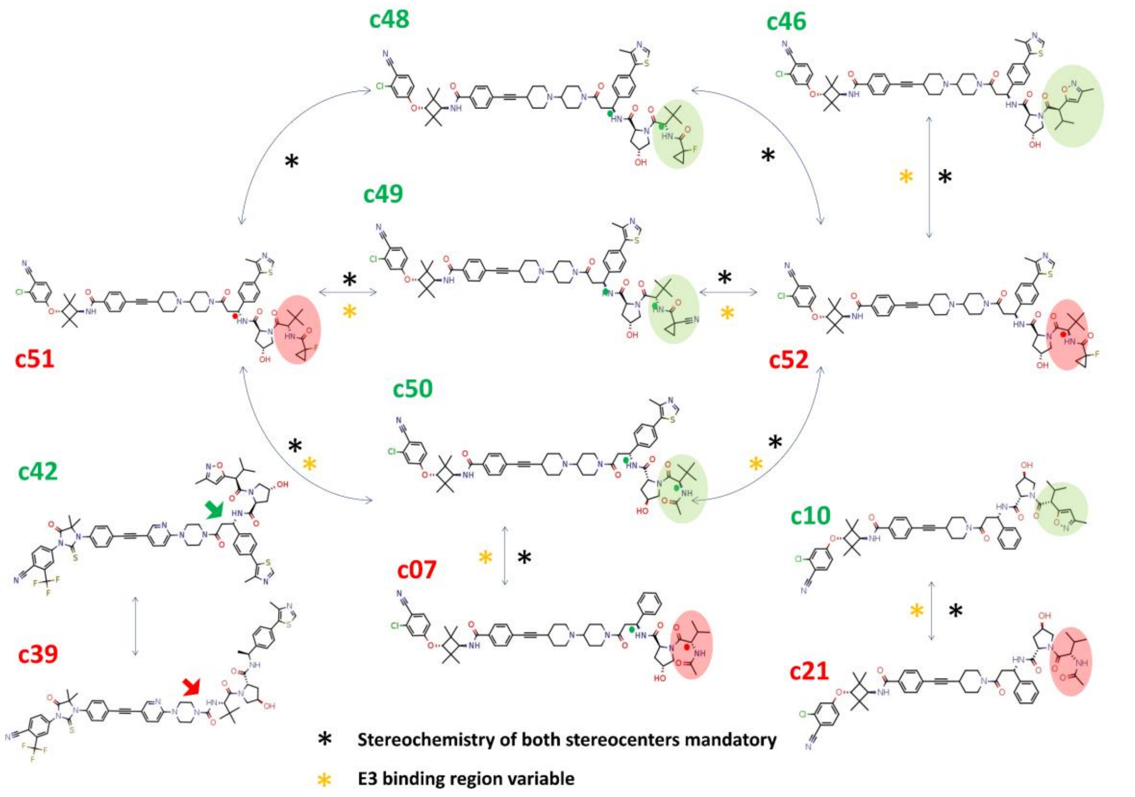

Figure S5. E3 ligand moiety-related Degradation Cliffs. The arrows connect compounds included in the same pair and the features responsible for the DC pair are highlighted in green (active compound related modification) or red (inactive compound related modification).

VHL c31, n= 11 (30)

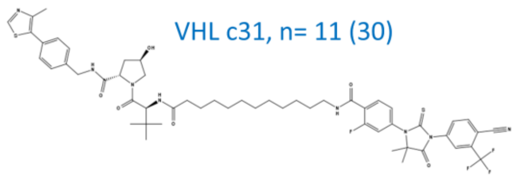

CRBN c64, n= 11 (39)

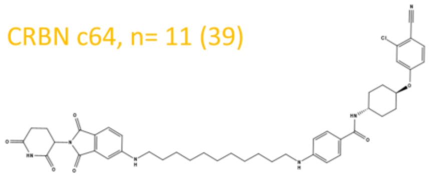

VHL c28, n= 8 (66)

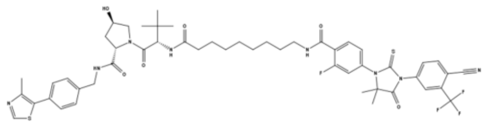

CRBN c59, n= 6 (86)

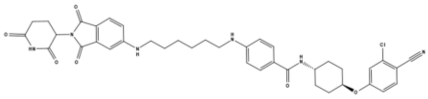

VHL c22, n= 2 (26)

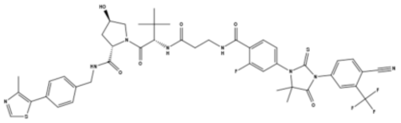

CRBN c56, n=3 (61)

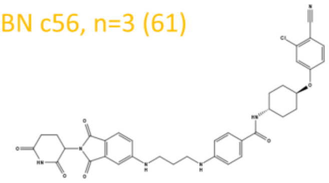

Name	Ligase	Linker	nC	Degradation
c22	VHL	linear	45	26
c23	VHL	linear	46	15
c24	VHL	linear	47	16
c25	VHL	linear	48	11
c26	VHL	linear	49	54
c27	VHL	linear	50	29
c28	VHL	linear	51	66
c29	VHL	linear	52	48
c30	VHL	linear	53	48
c31	VHL	linear	54	30
c56	CRBN	linear	36	61
c57	CRBN	linear	37	55
c58	CRBN	linear	38	82
c59	CRBN	linear	39	86
c60	CRBN	linear	40	62
c61	CRBN	linear	41	65
c62	CRBN	linear	42	58
c63	CRBN	linear	42	1
c64	CRBN	linear	44	39
c83	CRBN	rigid	44	87
c84	CRBN	rigid	44	93
c85	CRBN	rigid	43	99
c86	CRBN	rigid	42	86
c87	CRBN	rigid	41	89
c88	CRBN	rigid	42	95
c89	CRBN	rigid	41	88

CRBN c86, piperazine-methylene-azetidine (86)

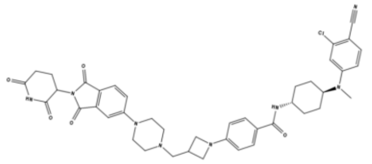

CRBN c85, piperazine-piperidine (99)

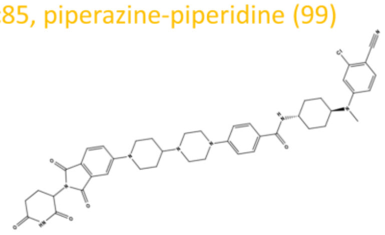

CRBN c83, piperazine-methylene-piperidine (87)

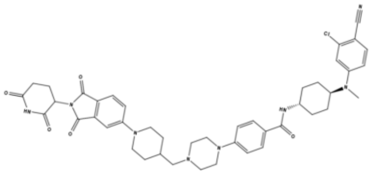

Figure S6. A table reporting degraders considered for linker analysis, the ligase recruited, the linker type, number of carbons per linker, and degradation data is shown. Three compounds are reported for each data series analyzed.

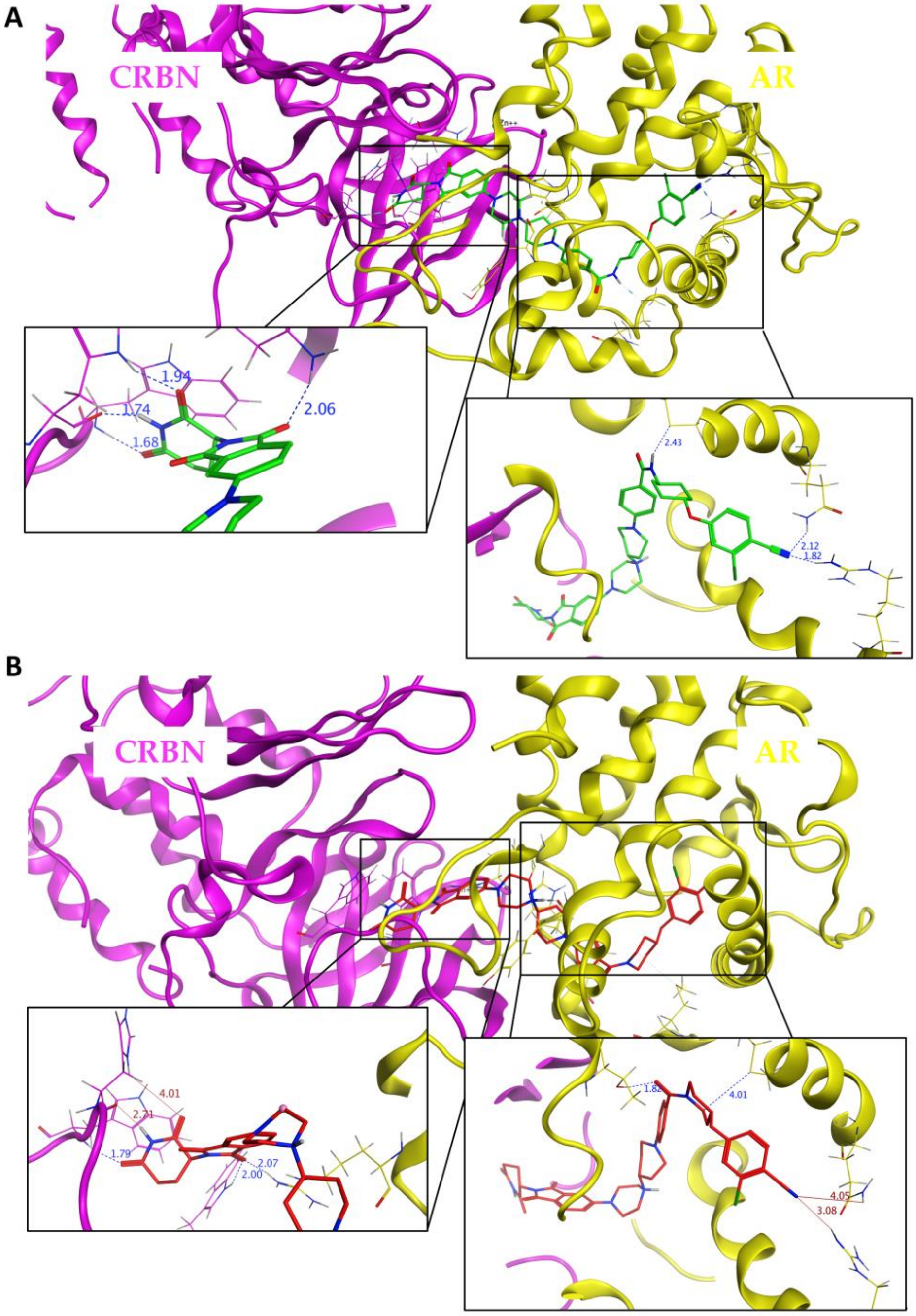

Figure S7. Comparison of c71 (active degrader) and c77 (inactive degrader) ternary complex. A) The ternary complex of c71 is shown. c71 is green-colored, AR is shown in yellow and CRBN in light violet; B) The ternary complex of c77 is shown. c77 is red-colored, AR is shown in yellow and CRBN in light violet. Hydrogen bonds are highlighted with blue dashed lines. Missing key interactions are highlighted with red lines.

A

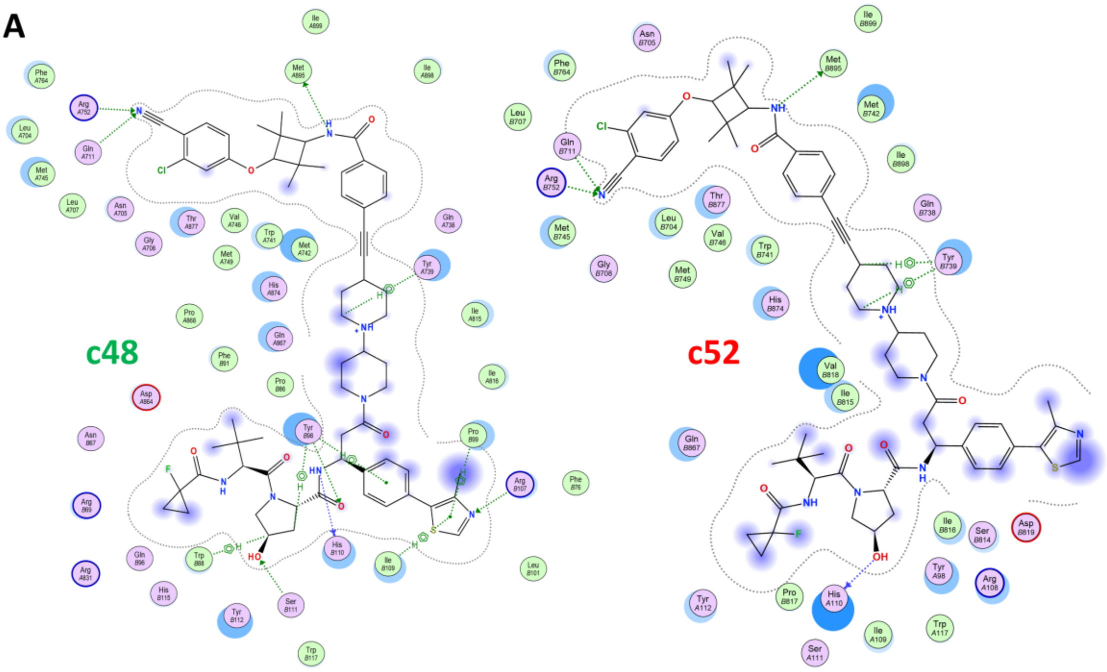

B

Ligand	Receptor	Interaction	Distance (A)	Energy (kcal/mol)
Cyano N	**Gln 711**, carboxamide	HB	3.07	-3.1
Piperidine ring	**Tyr 739**, phenol ring	H-pi	4.10	-0.8
Cyano N	**Arg 752**, guanidyl	HB	2.76	-7.3
Amidic N	**Met 895**, side chain S	HB	4.04	-1.5
Hydroxyproline ring	**Trp 88**, indole ring	H-Pi	3.90	-0.5
Amidic O	**Tyr 98**, hydroxyl	HB	2.78	-2.7
Benzene ring	**Tyr 98**, phenol ring	H-pi	3.84	-0.5
Hydroxyproline ring	**Tyr 98**, phenol ring	H-Pi	4.79	-0.5
Methyl-thiazole ring	**Pro 99**	H-pi	3.96	-0.7
Methyl-thiazole N	**Arg 107**, guanidyl	HB	2.99	-6.8
Methyl-thiazole ring	**Ile 109**	H-pi	3.95	-0.5
Amidic N	**His 110**, backbone O	HB	3.05	-2.4
Hydroxyl	**Ser 111**, hydroxyl	HB	2.94	-0.9
				**Total energy -28.2 kcal/mol**

C

Ligand	Receptor	Interaction	Distance (A)	Energy (kcal/mol)
Cyano N	**Gln 711**, carboxamide	HB	3.01	-3.9
Piperidine ring	**Tyr 739**, phenol ring	H-pi	3.90	-1.2
Piperidine ring	**Tyr 739**, phenol ring	H-pi	4.23	-0.6
Cyano N	**Arg 752**, guanidyl	HB	2.68	-6.0
Amidic N	**Met 895**	HB	3.98	-1.7
Hydroxyl	**His 110**, backbone O	HB	2.54	-1.8
				**Total energy -15.2 kcal/mol**

Figure S8. Comparison of c48 (active degrader) and c52 (inactive degrader). A) Ligand interaction chart of c48 and c52 are shown; B) c48 table of interactions with AR (yellow) and VHL (cyan) are reported; C) c52 table of interactions with AR (yellow) and VHL (cyan) are shown.

A

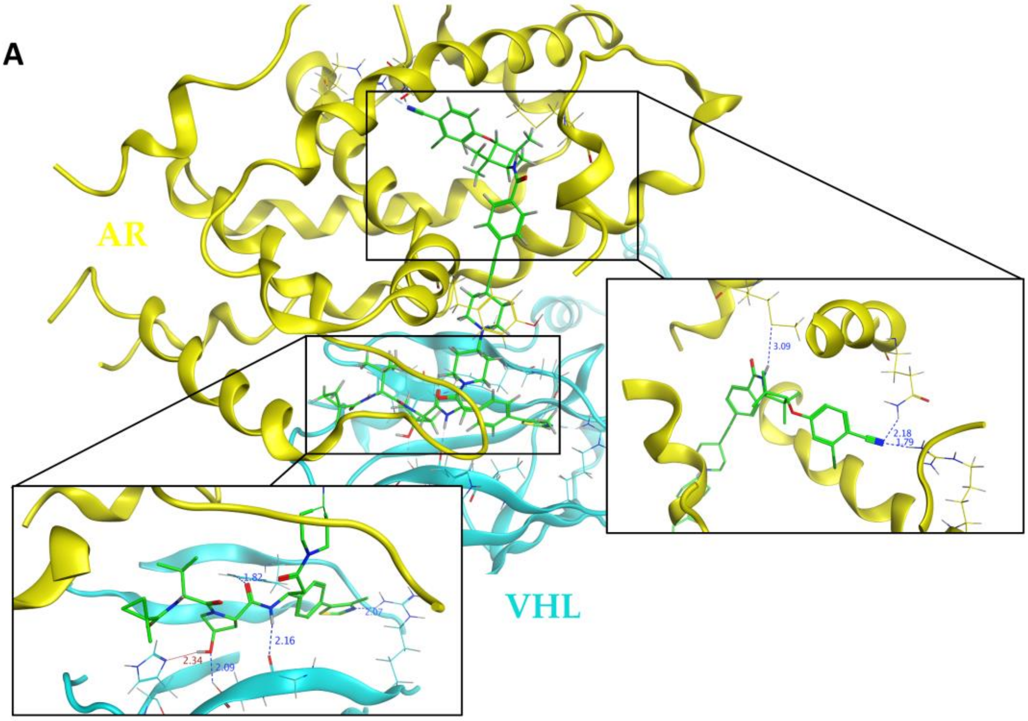

B

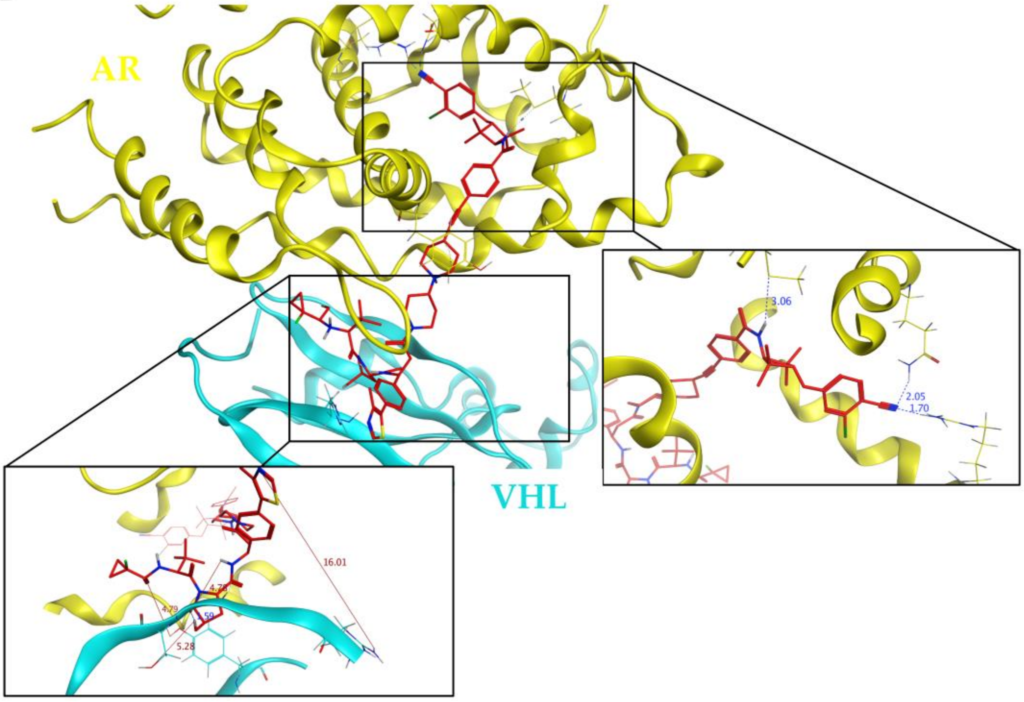

Figure S-9. Comparison of c48 (active degrader) and c52 (inactive degrader) ternary complex. A) The ternary complex of c48 is shown. c48 is green-colored, AR is shown in yellow and VHL in cyan; B) The ternary complex of c52 is shown. c52 is red-colored, AR is shown in yellow and VHL cyan.

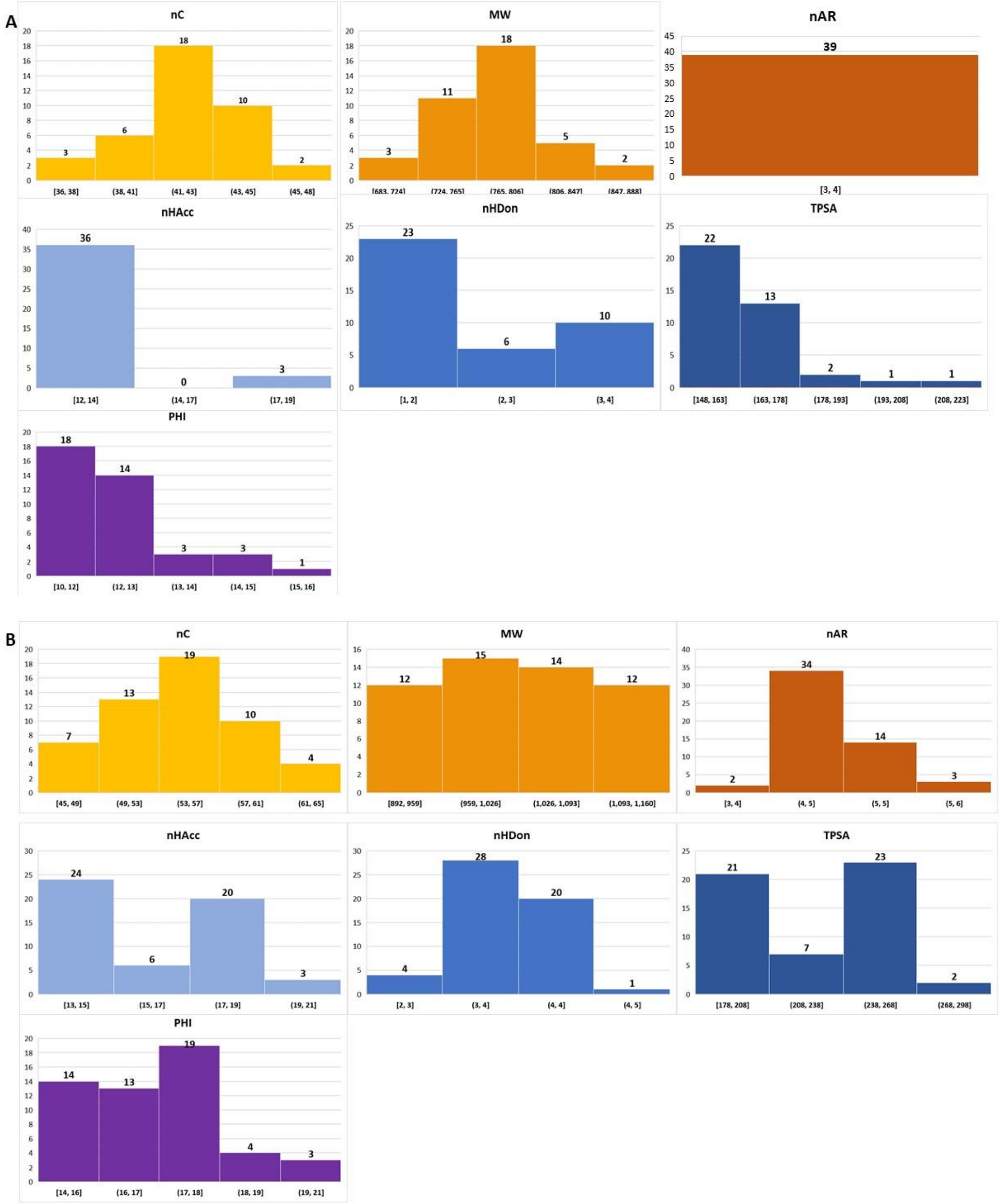

Figure S10. Distribution of the entire dataset for each descriptor is shown. In figure A the distribution of the molecular descriptors of CRBN-recruiting compounds is reported, in figure B VHL compounds. Histograms for non-polar descriptors are represented in shades of orange (nC, MW, nAR), polar descriptors (nHAcc, nHDon, TPSA) are shown in shades of blue, and PHI the flexibility descriptor is reported in violet.

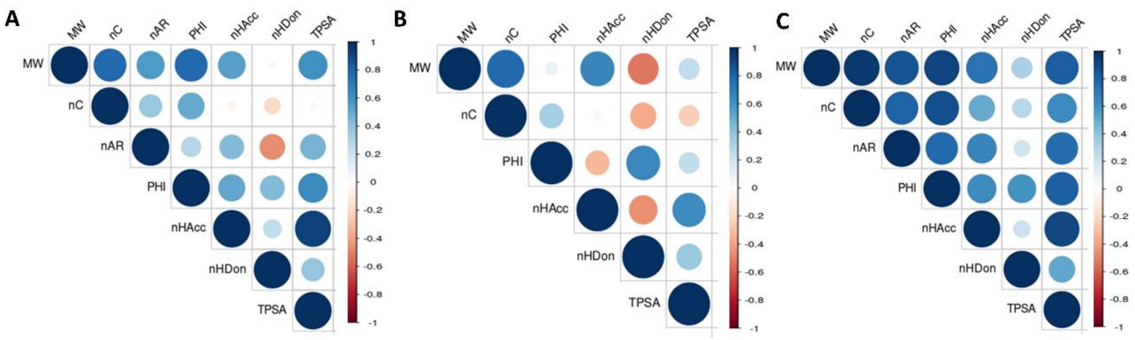

Figure S11. Correlation matrices respectively for VHL degraders figure A, CRBN figure B, and the entire dataset figure C.

Table S1: Table reporting for each PROTAC: a. our notation, b. original paper notation, SMILES string, and compound 2D structure.

a.	b.	Smiles	Compound structure
**c01**	2	C#CC1=CC=C([C@H](CC(=O)N2CCC(N3CCC(C#CC4=CC=C(C(=O)N[C@H]5C(C)(C)[C@H](OC6=CC=C(C#N)C(Cl)=C6)C5(C)C)C=C4)CC3)CC2)NC(=O)[C@@H]2C[C@@H](O)CN2C(=O)[C@@H](C2=CC(C)=NO2)C(C)C)C=C1	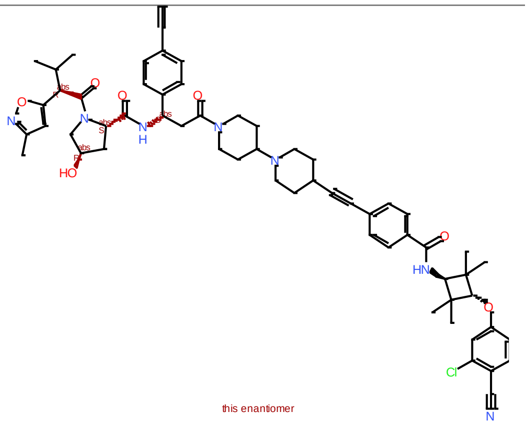
**c02**	3	CC1=NOC([C@H](C(=O)N2C[C@H](O)C[C@H]2C(=O)N[C@@H](CC(=O)N2CCC(N3CCC(C#CC4=CC=C(C(=O)N[C@H]5C(C)(C)[C@H](OC6=CC=C(C#N)C(Cl)=C6)C5(C)C)C=C4)CC3)CC2)C2=CC=C(C#N)C=C2)C(C)C)=C1	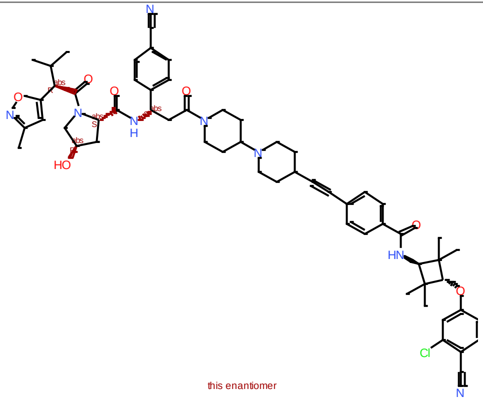
**c03**	4	CC1=NOC([C@H](C(=O)N2C[C@H](O)C[C@H]2C(=O)N[C@@H](CC(=O)N2CCC(N3CCC(C#CC4=CC=C(C(=O)N[C@H]5C(C)(C)[C@H](OC6=CC=C(C#N)C(Cl)=C6)C5(C)C)C=C4)CC3)CC2)C2=CC=C(Br)C=C2)C(C)C)=C1	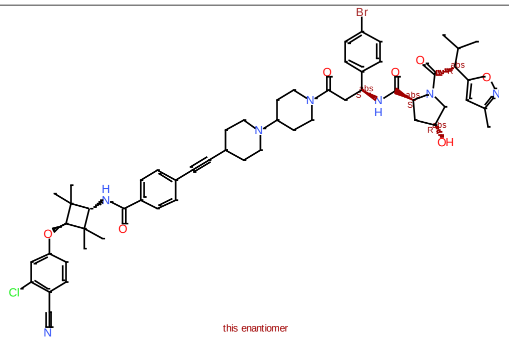

**c04**	5	CC1=NOC([C@H](C(=O)N2C[C@H](O)C[C@H]2C(=O)N[C@@H](CC(=O)N2CCC(N3CCC(C#CC4=CC=C(C(=O)N[C@H]5C(C)(C)[C@H](OC6=CC=C(C#N)C(Cl)=C6)C5(C)C)C=C4)CC3)CC2)C2=CC=C(Cl)C=C2)C(C)C)=C1	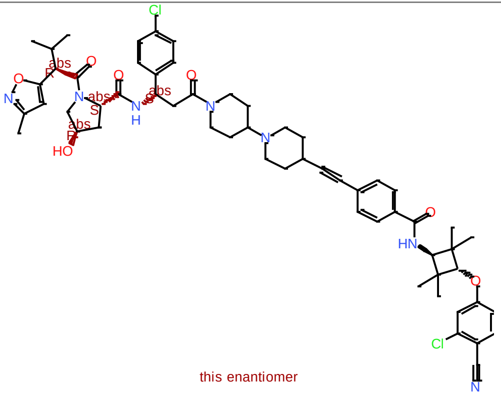
**c05**	6	CC1=NOC([C@H](C(=O)N2C[C@H](O)C[C@H]2C(=O)N[C@@H](CC(=O)N2CCC(N3CCC(C#CC4=CC=C(C(=O)N[C@H]5C(C)(C)[C@H](OC6=CC=C(C#N)C(Cl)=C6)C5(C)C)C=C4)CC3)CC2)C2=CC=C(F)C=C2)C(C)C)=C1	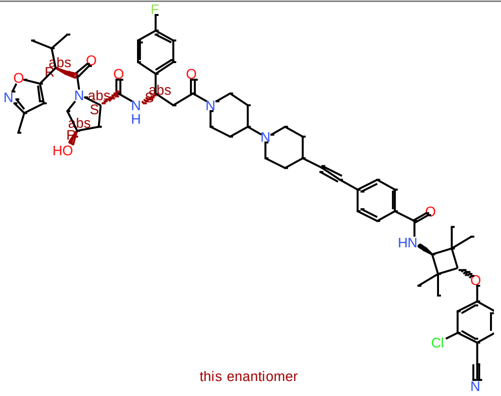
**c06**	7	CC1=NOC([C@H](C(=O)N2C[C@H](O)C[C@H]2C(=O)N[C@@H](CC(=O)N2CCC(N3CCC(C#CC4=CC=C(C(=O)N[C@H]5C(C)(C)[C@H](OC6=CC=C(C#N)C(Cl)=C6)C5(C)C)C=C4)CC3)CC2)C2=CC=CC=C2)C(C)C)=C1	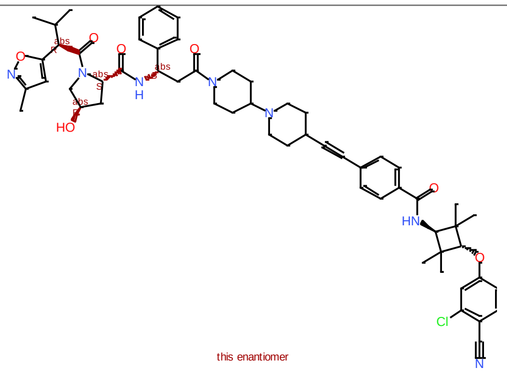
**c07**	8	CC(=O)N[C@H](C(=O)N1C[C@H](O)C[C@H]1C(=O)N[C@@H](CC(=O)N1CCC(N2CCC(C#CC3=CC=C(C(=O)N[C@H]4C(C)(C)[C@H](OC5=CC=C(C#N)C(Cl)=C5)C4(C)C)C=C3)CC2)CC1)C1=CC=CC=C1)C(C)C	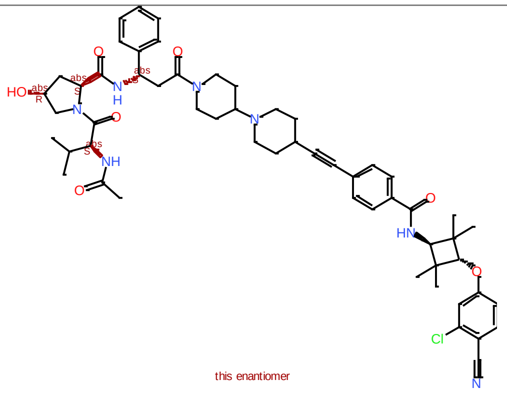

**c08**	9	CC1=NOC([C@H](C(=O)N2C[C@H](O)C[C@H]2C(=O)N[C@@H](CC(=O)N2CCC(N3CC(C#CC4=CC=C(C(=O)N[C@H]5C(C)(C)[C@H](OC6=CC=C(C#N)C(Cl)=C6)C5(C)C)C=C4)C3)CC2)C2=CC=CC=C2)C(C)C)=C1	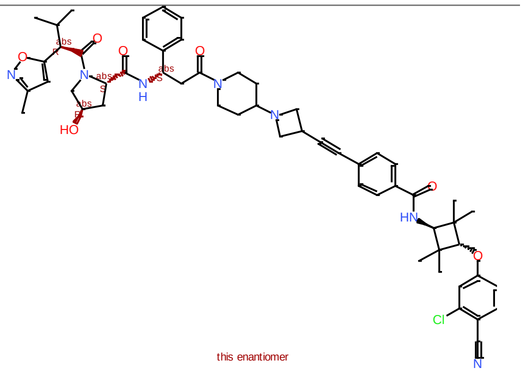
**c09**	10	CC1=NOC([C@H](C(=O)N2C[C@H](O)C[C@H]2C(=O)N[C@@H](CC(=O)N2CC(N3CCC(C#CC4=CC=C(C(=O)N[C@H]5C(C)(C)[C@H](OC6=CC=C(C#N)C(Cl)=C6)C5(C)C)C=C4)CC3)C2)C2=CC=CC=C2)C(C)C)=C1	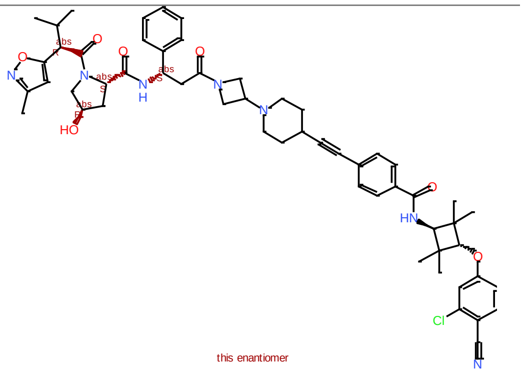
**c10**	11	CC1=NOC([C@H](C(=O)N2C[C@H](O)C[C@H]2C(=O)N[C@@H](CC(=O)N2CCC(C#CC3=CC=C(C(=O)N[C@H]4C(C)(C)[C@H](OC5=CC=C(C#N)C(Cl)=C5)C4(C)C)C=C3)CC2)C2=CC=CC=C2)C(C)C)=C1	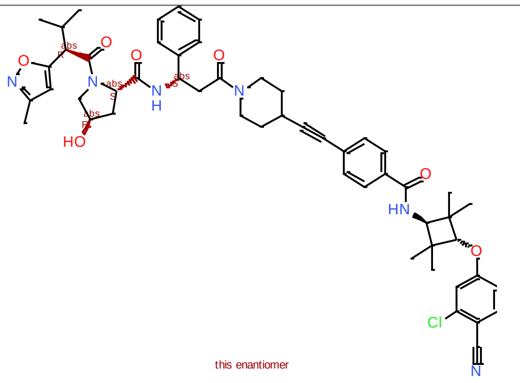
**c11**	12	CC1=NOC([C@H](C(=O)N2C[C@H](O)C[C@H]2C(=O)N[C@@H](CC(=O)N2CCC(C3CN(C4=CC=C(C(=O)N[C@H]5C(C)(C)[C@H](OC6=CC=C(C#N)C(Cl)=C6)C5(C)C)C=C4)C3)CC2)C2=CC=CC=C2)C(C)C)=C1	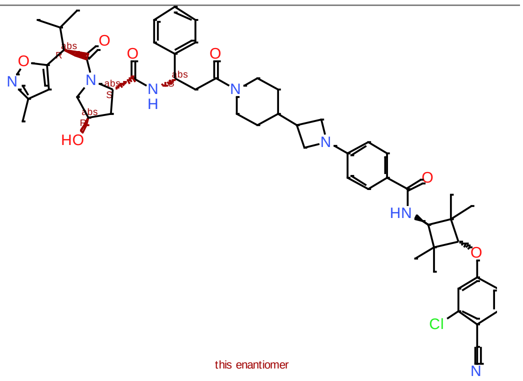

**c12**	13	CC1=NOC([C@H](C(=O)N2C[C@H](O)C[C@H]2C(=O)N[C@@H](CC(=O)N2CCC(N3CC(C4=CC=C(C(=O)N[C@H]5C(C)(C)[C@H](OC6=CC=C(C#N)C(Cl)=C6)C5(C)C)C=C4)C3)CC2)C2=CC=CC=C2)C(C)C)=C1	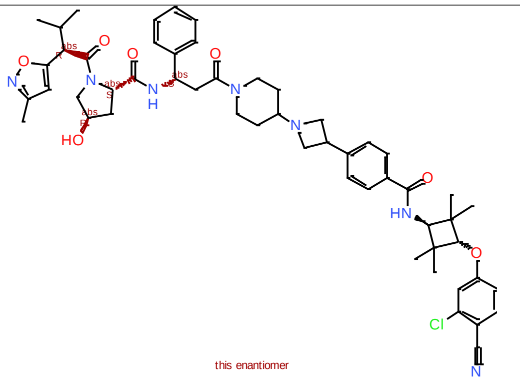
**c13**	14	CC1=NOC([C@H](C(=O)N2C[C@H](O)C[C@H]2C(=O)N[C@@H](CC(=O)N2CCC(C3=CC=C(C(=O)N[C@H]4C(C)(C)[C@H](OC5=CC=C(C#N)C(Cl)=C5)C4(C)C)C=C3)CC2)C2=CC=CC=C2)C(C)C)=C1	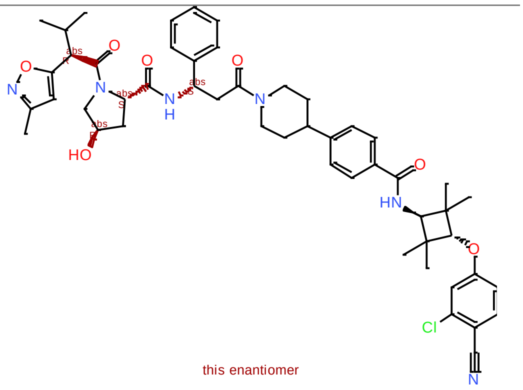
**c14**	15	CC1=NOC([C@H](C(=O)N2C[C@H](O)C[C@H]2C(=O)N[C@@H](CC(=O)N2CCN(C3=CC=C(C(=O)N[C@H]4C(C)(C)[C@H](OC5=CC=C(C#N)C(Cl)=C5)C4(C)C)C=C3)CC2)C2=CC=CC=C2)C(C)C)=C1	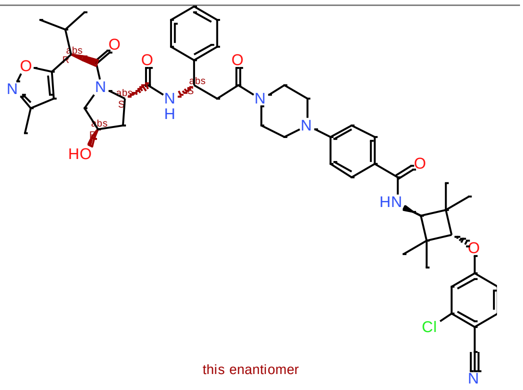
**c15**	16	CC1=NOC([C@H](C(=O)N2C[C@H](O)C[C@H]2C(=O)N[C@@H](CC(=O)N2CCC(C#CC3=CC=C(C(=O)N[C@H]4C(C)(C)[C@H](OC5=CC=C(C#N)C(Cl)=C5)C4(C)C)C=C3)CC2)C2=CC=C(C3=C(C)N=CS3)C=C2)C(C)C)=C1	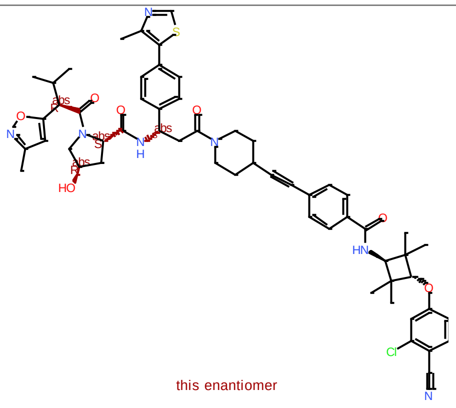

**c16**	17	CC1=NOC([C@H](C(=O)N2C[C@H](O)C[C@H]2C(=O)N[C@@H](CC(=O)N2CCC(C#CC3=CC=C(C(=O)N[C@H]4C(C)(C)[C@H](OC5=CC=C(C#N)C(Cl)=C5)C4(C)C)C=C3)CC2)C2=CC=C(F)C=C2)C(C)C)=C1	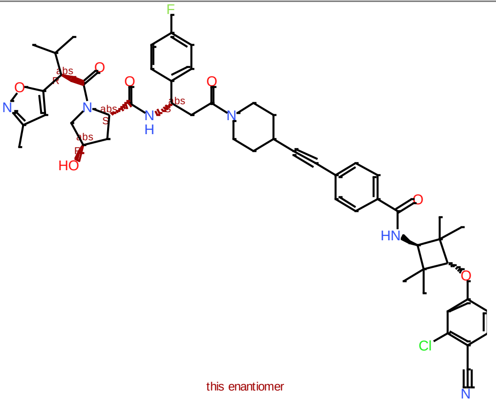
**c17**	18	CC1=NOC([C@H](C(=O)N2C[C@H](O)C[C@H]2C(=O)N[C@@H](CC(=O)N2CCC(C#CC3=CC=C(C(=O)N[C@H]4C(C)(C)[C@H](OC5=CC=C(C#N)C(Cl)=C5)C4(C)C)C=C3)CC2)C2=CC=C(Cl)C=C2)C(C)C)=C1	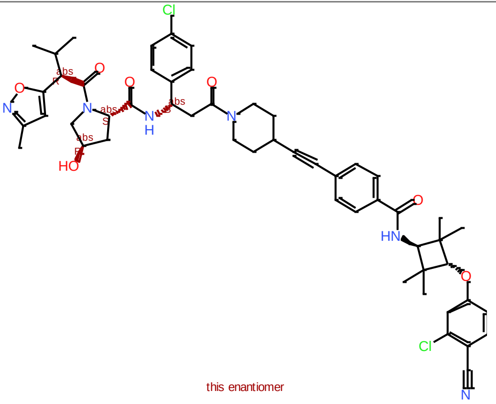
**c18**	19	CC1=NOC([C@H](C(=O)N2C[C@H](O)C[C@H]2C(=O)N[C@@H](CC(=O)N2CCC(C#CC3=CC=C(C(=O)N[C@H]4C(C)(C)[C@H](OC5=CC=C(C#N)C(Cl)=C5)C4(C)C)C=C3)CC2)C2=CC=C(Br)C=C2)C(C)C)=C1	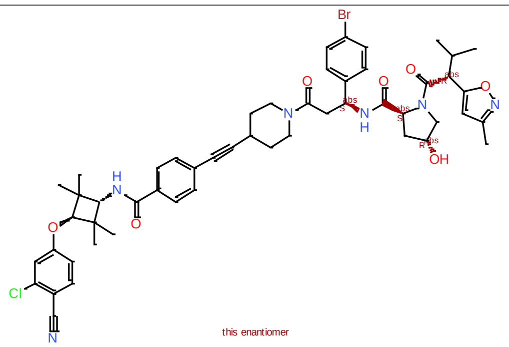
**c19**	20	CC1=NOC([C@H](C(=O)N2C[C@H](O)C[C@H]2C(=O)N[C@@H](CC(=O)N2CCC(C#CC3=CC=C(C(=O)N[C@H]4C(C)(C)[C@H](OC5=CC=C(C#N)C(Cl)=C5)C4(C)C)C=C3)CC2)C2=CC=C(C#N)C=C2)C(C)C)=C1	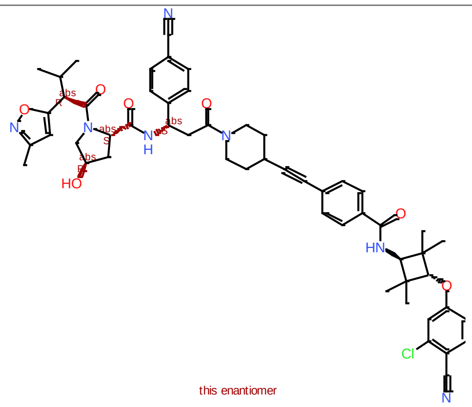

**c20**	21	C#CC1=CC=C([C@H](CC(=O)N2CCC(C#CC3=CC=C(C(=O)N[C@H]4C(C)(C)[C@H](OC5=CC=C(C#N)C(Cl)=C5)C4(C)C)C=C3)CC2)NC(=O)[C@@H]2C[C@@H](O)CN2C(=O)[C@@H](C2=CC(C)=NO2)C(C)C)C=C1	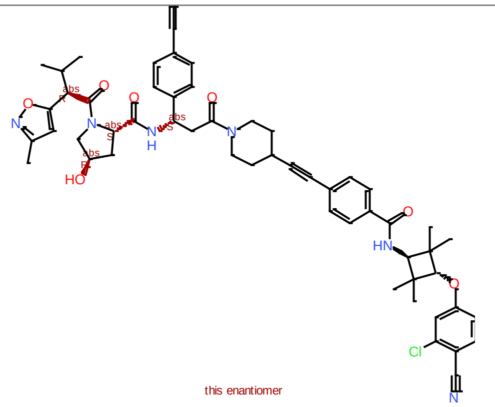
**c21**	22	CC(=O)N[C@H](C(=O)N1C[C@H](O)C[C@H]1C(=O)N[C@@H](CC(=O)N1CCC(C#CC2=CC=C(C(=O)N[C@H]3C(C)(C)[C@H](OC4=CC=C(C#N)C(Cl)=C4)C3(C)C)C=C2)CC1)C1=CC=CC=C1)C(C)C	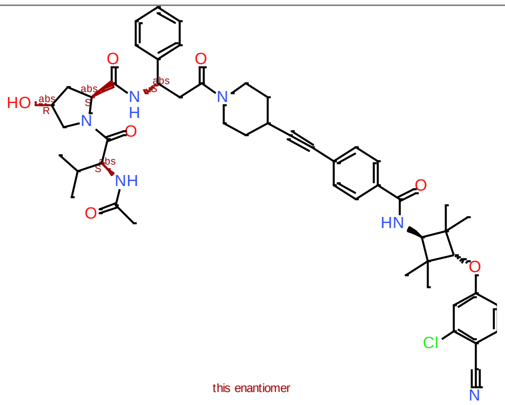
**c22**	8	CC1=C(C2=CC=C(CNC(=O)[C@@H]3C[C@@H](O)CN3C(=O)[C@@H](NC(=O)CCNC(=O)C3=CC=C(N4C(=S)N(C5=CC=C(C#N)C(C(F)(F)F)=C5)C(=O)C4(C)C)C=C3F)C(C)(C)C)C=C2)SC=N1	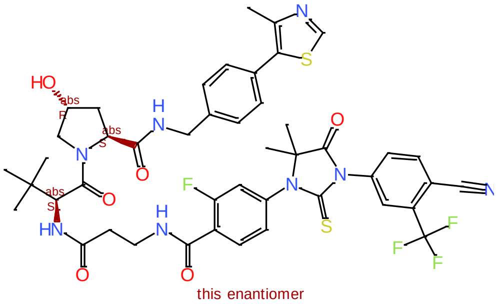
**c23**	9	CC1=C(C2=CC=C(CNC(=O)[C@@H]3C[C@@H](O)CN3C(=O)[C@@H](NC(=O)CCCNC(=O)C3=CC=C(N4C(=S)N(C5=CC=C(C#N)C(C(F)(F)F)=C5)C(=O)C4(C)C)C=C3F)C(C)(C)C)C=C2)SC=N1	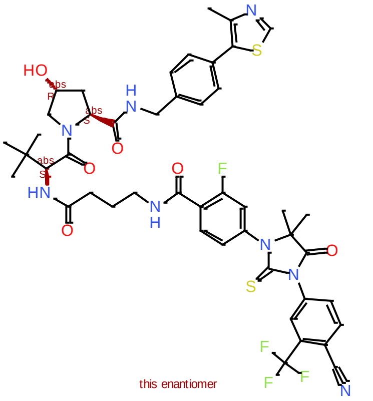

**c24**	10	CC1=C(C2=CC=C(CNC(=O)[C@@H]3C[C@@H](O)CN3C(=O)[C@@H](NC(=O)CCCCNC(=O)C3=CC=C(N4C(=S)N(C5=CC=C(C#N)C(C(F)(F)F)=C5)C(=O)C4(C)C)C=C3F)C(C)(C)C)C=C2)SC=N1	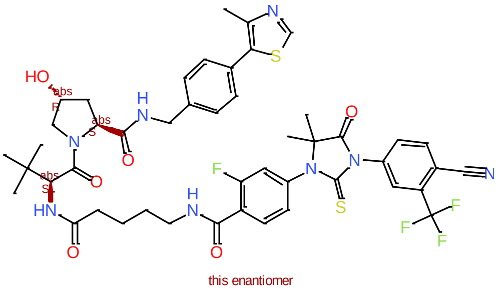
**c25**	11	CC1=C(C2=CC=C(CNC(=O)[C@@H]3C[C@@H](O)CN3C(=O)[C@@H](NC(=O)CCCCCNC(=O)C3=CC=C(N4C(=S)N(C5=CC=C(C#N)C(C(F)(F)F)=C5)C(=O)C4(C)C)C=C3F)C(C)(C)C)C=C2)SC=N1	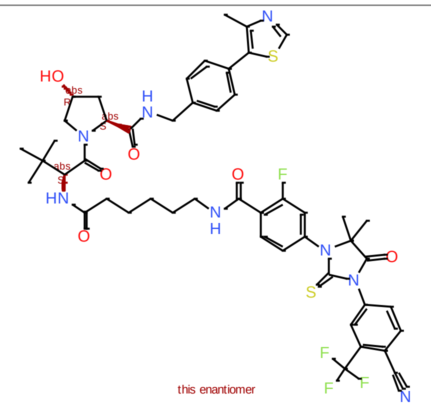
**c26**	12	CC1=C(C2=CC=C(CNC(=O)[C@@H]3C[C@@H](O)CN3C(=O)[C@@H](NC(=O)CCCCCCNC(=O)C3=CC=C(N4C(=S)N(C5=CC=C(C#N)C(C(F)(F)F)=C5)C(=O)C4(C)C)C=C3F)C(C)(C)C)C=C2)SC=N1	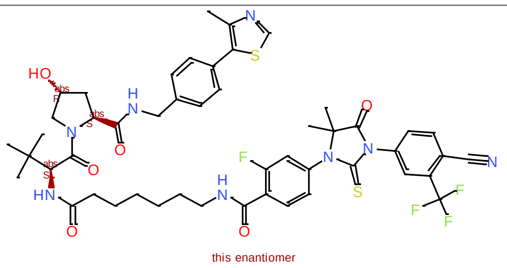
**c27**	13	CC1=C(C2=CC=C(CNC(=O)[C@@H]3C[C@@H](O)CN3C(=O)[C@@H](NC(=O)CCCCCCCNC(=O)C3=CC=C(N4C(=S)N(C5=CC=C(C#N)C(C(F)(F)F)=C5)C(=O)C4(C)C)C=C3F)C(C)(C)C)C=C2)SC=N1	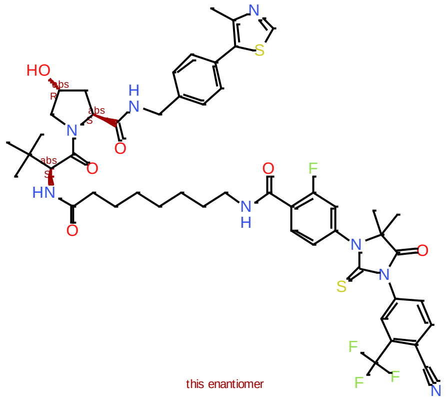
**c28**	14	CC1=C(C2=CC=C(CNC(=O)[C@@H]3C[C@@H](O)CN3C(=O)[C@@H](NC(=O)CCCCCCCCNC(=O)C3=CC=C(N4C(=S)N(C5=CC=C(C#N)C(C(F)(F)F)=C5)C(=O)C4(C)C)C=C3F)C(C)(C)C)C=C2)SC=N1	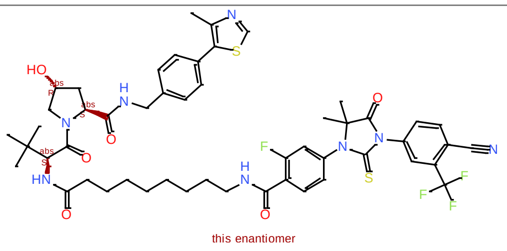

**c29**	15	CC1=C(C2=CC=C(CNC(=O)[C@@H]3C[C@@H](O)CN3C(=O)[C@@H](NC(=O)CCCCCCCCNC(=O)C3=CC=C(N4C(=S)N(C5=CC=C(C#N)C(C(F)(F)F)=C5)C(=O)C4(C)C)C=C3F)C(C)(C)C)C=C2)SC=N1	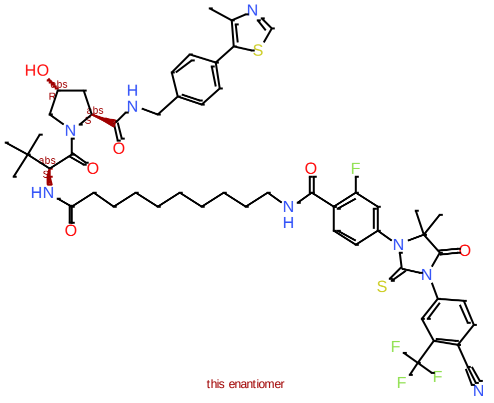
**c30**	16	CC1=C(C2=CC=C(CNC(=O)[C@@H]3C[C@@H](O)CN3C(=O)[C@@H](NC(=O)CCCCCCCCCNC(=O)C3=CC=C(N4C(=S)N(C5=CC=C(C#N)C(C(F)(F)F)=C5)C(=O)C4(C)C)C=C3F)C(C)(C)C)C=C2)SC=N1	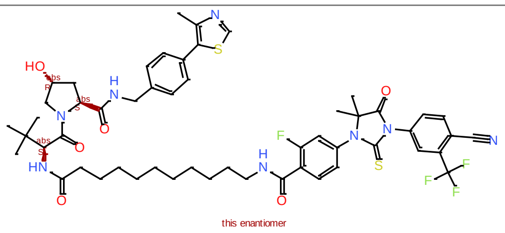
**c31**	17	CC1=C(C2=CC=C(CNC(=O)[C@@H]3C[C@@H](O)CN3C(=O)[C@@H](NC(=O)CCCCCCCCCCNC(=O)C3=CC=C(N4C(=S)N(C5=CC=C(C#N)C(C(F)(F)F)=C5)C(=O)C4(C)C)C=C3F)C(C)(C)C)C=C2)SC=N1	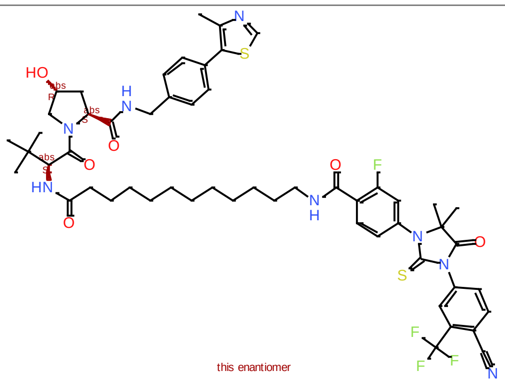
**c32**	18	CC1=C(C2=CC=C(CNC(=O)[C@@H]3C[C@@H](O)CN3C(=O)[C@@H](NC(=O)CCOCCOCCNC(=O)C3=CC=C(N4C(=S)N(C5=CC=C(C#N)C(C(F)(F)F)=C5)C(=O)C4(C)C)C=C3F)C(C)(C)C)C=C2)SC=N1	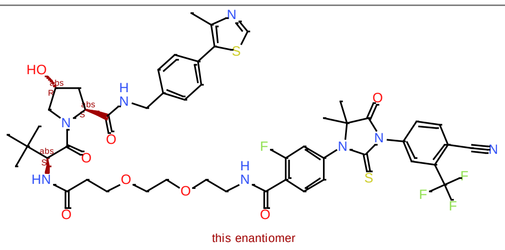
**c33**	19	CC1=C(C2=CC=C(CNC(=O)[C@@H]3C[C@@H](O)CN3C(=O)[C@@H](NC(=O)CCCCCCC3=CC=C(C#CC4=CC=C(N5C(=S)N(C6=CC=C(C#N)C(C(F)(F)F)=C6)C(=O)C5(C)C)C=C4)C=N3)C(C)(C)C)C=C2)SC=N1	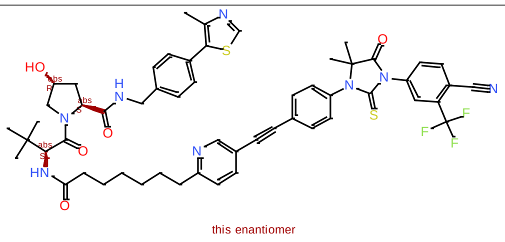

**c34**	20	CC1=C(C2=CC=C(CNC(=O)[C@@H]3C[C@@H](O)CN3C(=O)[C@@H](NC(=O)CCCN3CCN(C4=CC=C(C#CC5=CC=C(N6C(=S)N(C7=CC=C(C#N)C(C(F)(F)F)=C7)C(=O)C6(C)C)C=C5)C=N4)CC3)C(C)(C)C)C=C2)SC=N1	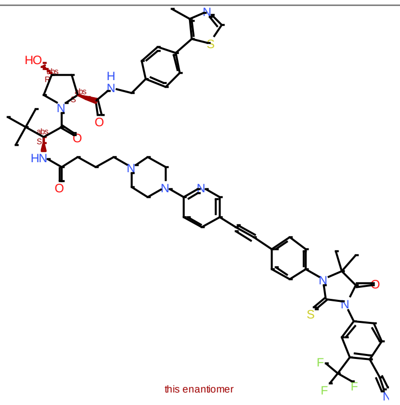
**c35**	21	CC1=C(C2=CC=C([C@H](C)NC(=O)[C@@H]3C[C@@H](O)CN3C(=O)[C@@H](NC(=O)CCCN3CCN(C4=CC=C(C#CC5=CC=C(N6C(=S)N(C7=CC=C(C#N)C(C(F)(F)F)=C7)C(=O)C6(C)C)C=C5)C=N4)CC3)C(C)(C)C)C=C2)SC=N1	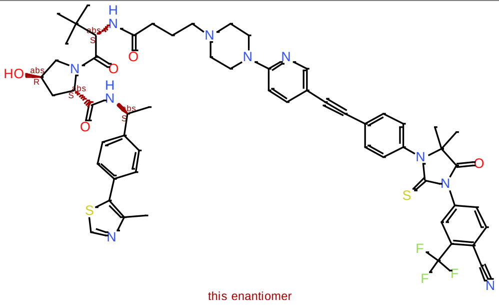
**c36**	22	CC1=C(C2=CC=C([C@H](C)NC(=O)[C@@H]3C[C@@H](O)CN3C(=O)[C@@H](NC(=O)CCCCN3CCN(C4=CC=C(C#CC5=CC=C(N6C(=S)N(C7=CC=C(C#N)C(C(F)(F)F)=C7)C(=O)C6(C)C)C=C5)C=N4)CC3)C(C)(C)C)C=C2)SC=N1	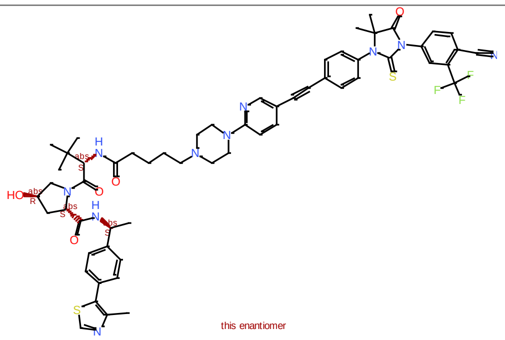
**c37**	23	CC1=C(C2=CC=C([C@H](C)NC(=O)[C@@H]3C[C@@H](O)CN3C(=O)[C@@H](NC(=O)CCN3CCN(C4=CC=C(C#CC5=CC=C(N6C(=S)N(C7=CC=C(C#N)C(C(F)(F)F)=C7)C(=O)C6(C)C)C=C5)C=N4)CC3)C(C)(C)C)C=C2)SC=N1	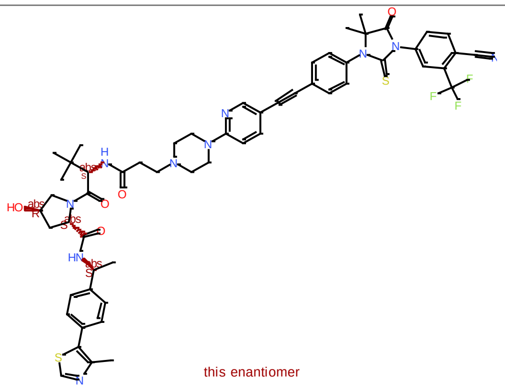

**c38**	24	CC1=C(C2=CC=C([C@H](C)NC(=O)[C@@H]3C[C@@H](O)CN3C(=O)[C@@H](NC(=O)CN3CCN(C4=CC=C(C#CC5=CC=C(N6C(=S)N(C7=CC=C(C#N)C(C(F)(F)F)=C7)C(=O)C6(C)C)C=C5)C=N4)CC3)C(C)(C)C)C=C2)SC=N1	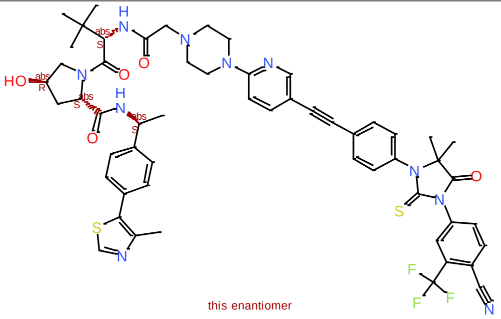
**c39**	25	CC1=C(C2=CC=C([C@H](C)NC(=O)[C@@H]3C[C@@H](O)CN3C(=O)[C@@H](NC(=O)N3CCN(C4=CC=C(C#CC5=CC=C(N6C(=S)N(C7=CC=C(C#N)C(C(F)(F)F)=C7)C(=O)C6(C)C)C=C5)C=N4)CC3)C(C)(C)C)C=C2)SC=N1	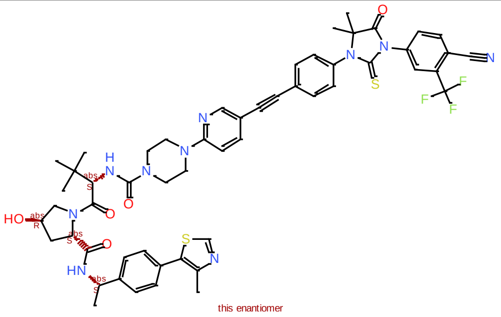
**c40**	26	CC1=NOC([C@H](C(=O)N2C[C@H](O)C[C@H]2C(=O)N[C@@H](CC(=O)NCCCCCCCCCCNC(=O)C2=CC=C(N3C(=S)N(C4=CC=C(C#N)C(C(F)(F)F)=C4)C(=O)C3(C)C)C=C2F)C2=CC=C(C3=C(C)N=CS3)C=C2)C(C)C)=C1	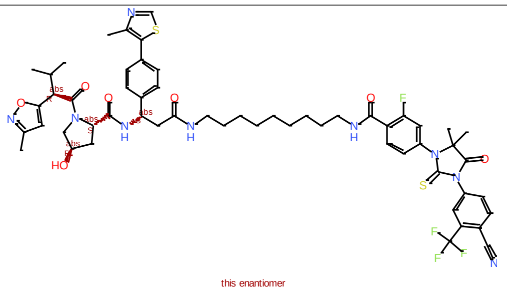
**c41**	27	CC1=NOC([C@H](C(=O)N2C[C@H](O)C[C@H]2C(=O)N[C@@H](CC(=O)NCCCN2CCN(C3=CC=C(C#CC4=CC=C(N5C(=S)N(C6=CC=C(C#N)C(C(F)(F)F)=C6)C(=O)C5(C)C)C=C4)C=N3)CC2)C2=CC=C(C3=C(C)N=CS3)C=C2)C(C)C)=C1	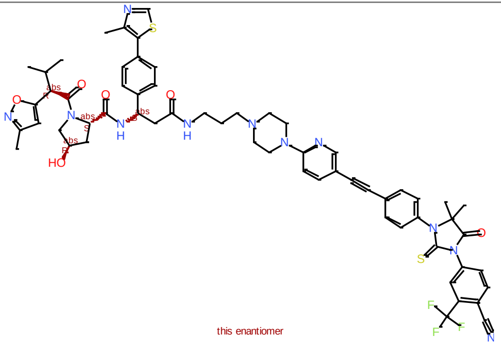

**c42**	28	CC1=NOC([C@H](C(=O)N2C[C@H](O)C[C@H]2C(=O)N[C@@H](CC(=O)N2CCN(C3=CC=C(C#CC4=CC=C(N5C(=S)N(C6=CC=C(C#N)C(C(F)(F)F)=C6)C(=O)C5(C)C)C=C4)C=N3)CC2)C2=CC=C(C3=C(C)N=CS3)C=C2)C(C)C)=C1	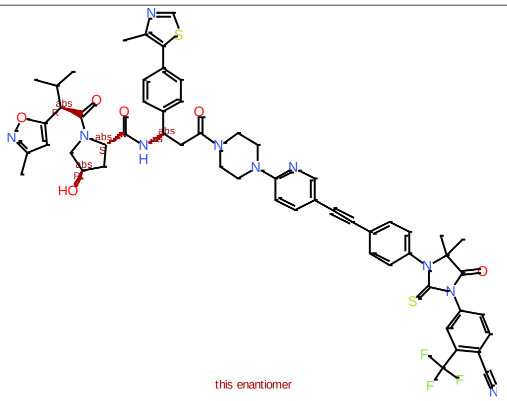
**c43**	29	CC1=NOC([C@H](C(=O)N2C[C@H](O)C[C@H]2C(=O)N[C@@H](CC(=O)N2CCC(N3CCC(C#CC4=CC=C(N5C(=S)N(C6=CC=C(C#N)C(C(F)(F)F)=C6)C(=O)C5(C)C)C=C4)CC3)CC2)C2=CC=C(C3=C(C)N=CS3)C=C2)C(C)C)=C1	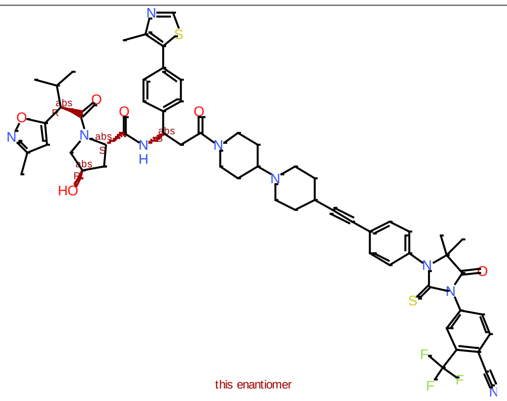
**c44**	30	CC1=NOC([C@H](C(=O)N2C[C@H](O)C[C@H]2C(=O)N[C@@H](CC(=O)N2CCC(N3CCC(C#CC4=CC=C(N5C(=S)N(C6=CN=C(C#N)C(C(F)(F)F)=C6)C(=O)C56CCC6)C=C4)CC3)CC2)C2=CC=C(C3=C(C)N=CS3)C=C2)C(C)C)=C1	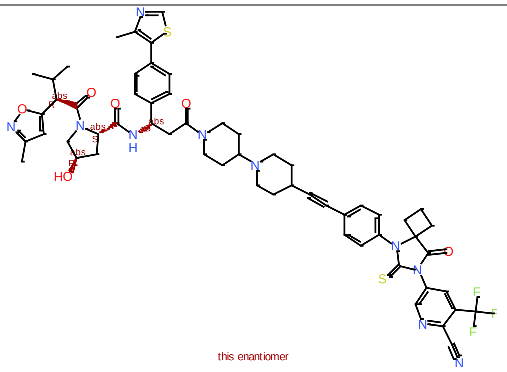
**c45**	31	CC1=NOC([C@H](C(=O)N2C[C@H](O)C[C@H]2C(=O)N[C@@H](CC(=O)N2CCC(N3CCC(C#CC4=CC=C(S(=O)CC(C)(O)C(=O)NC5=CC=C(C#N)C(C(F)(F)F)=C5)C=C4)CC3)CC2)C2=CC=C(C3=C(C)N=CS3)C=C2)C(C)C)=C1	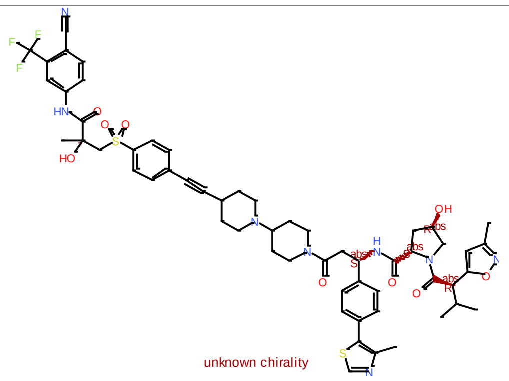

**c46**	32	CC1=NOC([C@H](C(=O)N2C[C@H](O)C[C@H]2C(=O)N[C@@H](CC(=O)N2CCC(N3CCC(C#CC4=CC=C(C(=O)N[C@H]5C(C)(C)[C@H](OC6=CC=C(C#N)C(Cl)=C6)C5(C)C)C=C4)CC3)CC2)C2=CC=C(C3=C(C)N=CS3)C=C2)C(C)C)=C1	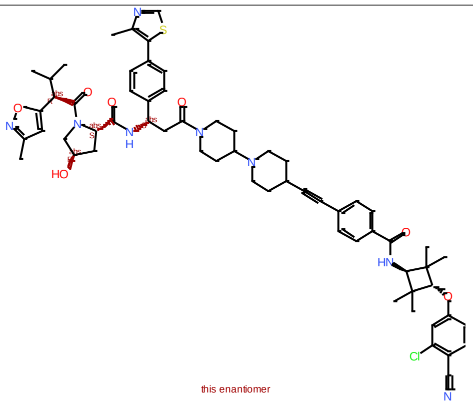
**c47**	33	CC1=NOC([C@H](C(=O)N2C[C@H](O)C[C@H]2C(=O)N[C@@H](CC(=O)N2CCC(N3CCC(C#CC4=CC=C(CC5=C(C)N(C6=CC=C(C#N)C(Cl)=C6)N=C5C)C=C4)CC3)CC2)C2=CC=C(C3=C(C)N=CS3)C=C2)C(C)C)=C1	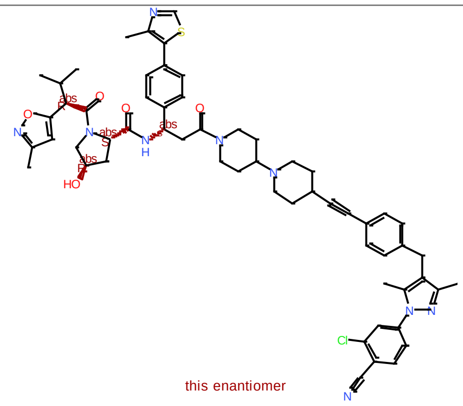
**c48**	34	CC1=C(C2=CC=C([C@H](CC(=O)N3CCC(N4CCC(C#CC5=CC=C(C(=O)N[C@H]6C(C)(C)[C@H](OC7=CC=C(C#N)C(Cl)=C7)C6(C)C)C=C5)CC4)CC3)NC(=O)[C@@H]3C[C@@H](O)CN3C(=O)[C@@H](NC(=O)C3(F)CC3)C(C)(C)C)C=C2)SC=N1	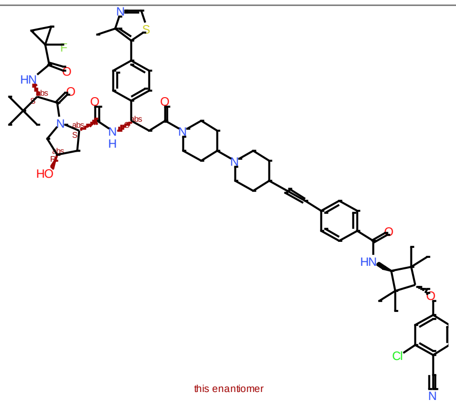
**c49**	35	CC1=C(C2=CC=C([C@H](CC(=O)N3CCC(N4CCC(C#CC5=CC=C(C(=O)N[C@H]6C(C)(C)[C@H](OC7=CC=C(C#N)C(Cl)=C7)C6(C)C)C=C5)CC4)CC3)NC(=O)[C@@H]3C[C@@H](O)CN3C(=O)[C@@H](NC(=O)C3(C#N)CC3)C(C)(C)C)C=C2)SC=N1	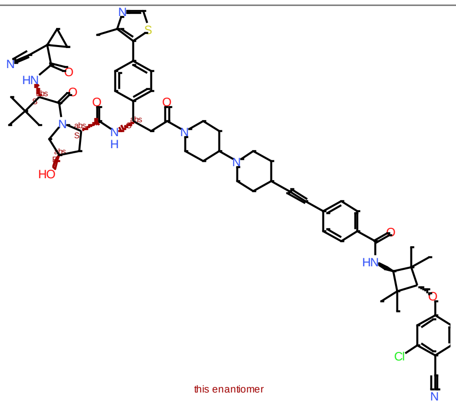

**c50**	36	CC(=O)N[C@H](C(=O)N1C[C@H](O)C[C@H]1C(=O)N[C@@H](CC(=O)N1CCC(N2CCC(C#CC3=CC=C(C(=O)N[C@H]4C(C)(C)[C@H](OC5=CC=C(C#N)C(Cl)=C5)C4(C)C)C=C3)CC2)CC1)C1=CC=C(C2=C(C)N=CS2)C=C1)C(C)(C)C	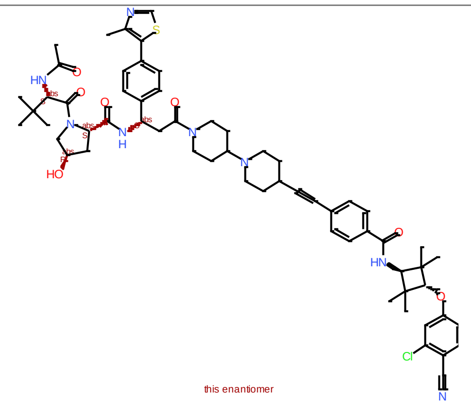
**c51**	37	CC1=C(C2=CC=C([C@@H](CC(=O)N3CCC(N4CCC(C#CC5=CC=C(C(=O)N[C@H]6C(C)(C)[C@H](OC7=CC=C(C#N)C(Cl)=C7)C6(C)C)C=C5)CC4)CC3)NC(=O)[C@@H]3C[C@@H](O)CN3C(=O)[C@@H](NC(=O)C3(F)CC3)C(C)(C)C)C=C2)SC=N1	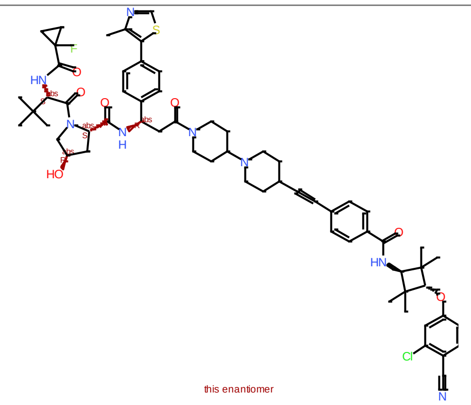
**c52**	38	CC1=C(C2=CC=C([C@H](CC(=O)N3CCC(N4CCC(C#CC5=CC=C(C(=O)N[C@H]6C(C)(C)[C@H](OC7=CC=C(C#N)C(Cl)=C7)C6(C)C)C=C5)CC4)CC3)NC(=O)[C@@H]3C[C@@H](O)CN3C(=O)[C@H](NC(=O)C3(F)CC3)C(C)(C)C)C=C2)SC=N1	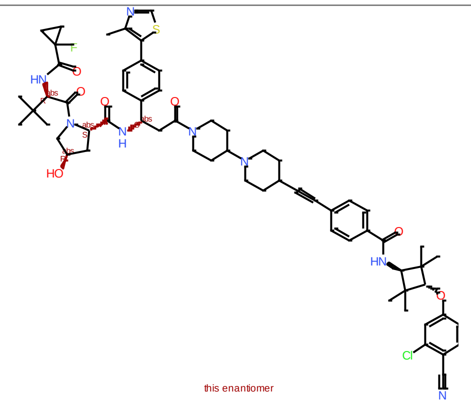
**c53**	39	CC1=NOC([C@H](C(=O)N2C[C@H](O)C[C@H]2C(=O)N[C@@H](CC(=O)NCCCCCCCCCNC(=O)C2=CC=C(C(=O)N[C@H]3C(C)(C)[C@H](OC4=CC=C(C#N)C(Cl)=C4)C3(C)C)C=C2)C2=CC=C(C3=C(C)N=CS3)C=C2)C(C)C)=C1	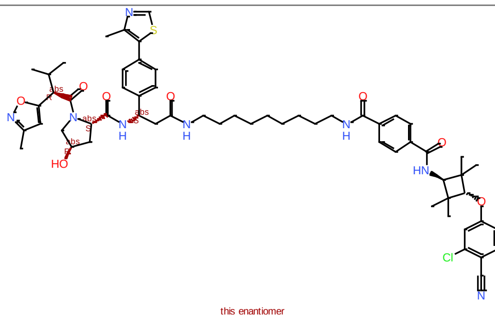

**c54**	40	CC1(C)[C@H](NC(=O)C2=CC=C(C(=O)NCCCCCCCCCCNC3=CC=CC4=C3C(=O)N(C3CCC(=O)NC3=O)C4=O)C=C2)C(C)(C)[C@H]1OC1=CC=C(C#N)C(Cl)=C1	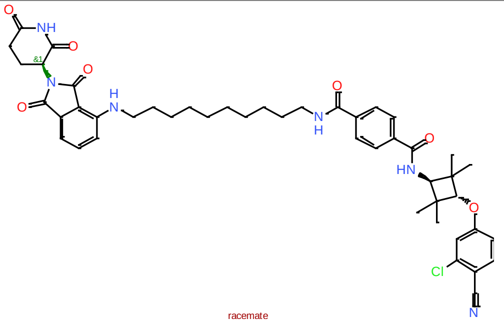
**c55**	41	CC1(C)[C@H](NC(=O)C2=CC=C(C#CC3CCN(C4CCN(C5=CC=CC6=C5C(=O)N(C5CCC(=O)NC5=O)C6=O)CC4)CC3)C=C2)C(C)(C)[C@H]1OC1=CC=C(C#N)C(Cl)=C1	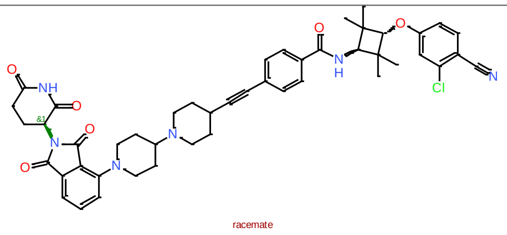
**c56**	11	ClC1=C(C#N)C=CC(O[C@@H](CC2)CC[C@H]2NC(C3=CC=C(NCCCNC4=CC5=C(C(N(C6C(NC(CC6)=O)=O)C5=O)=O)C=C4)C=C3)=O)=C1	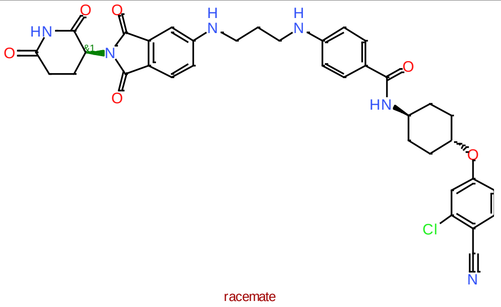
**c57**	12	ClC1=C(C#N)C=CC(O[C@@H](CC2)CC[C@H]2NC(C3=CC=C(NCCCCNC4=CC5=C(C(N(C6C(NC(CC6)=O)=O)C5=O)=O)C=C4)C=C3)=O)=C1	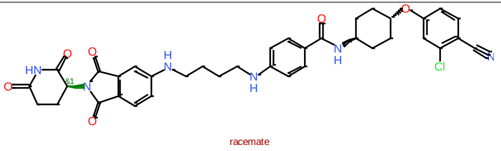
**c58**	13	ClC1=C(C#N)C=CC(O[C@@H](CC2)CC[C@H]2NC(C3=CC=C(NCCCCCNC4=CC5=C(C(N(C6C(NC(CC6)=O)=O)C5=O)=O)C=C4)C=C3)=O)=C1	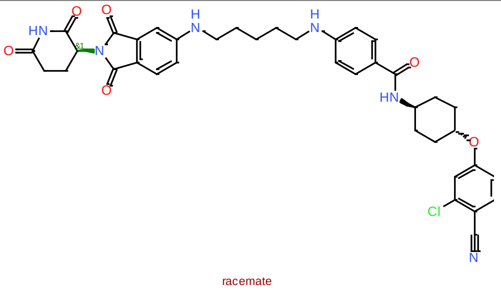
**c59**	14	ClC1=C(C#N)C=CC(O[C@@H](CC2)CC[C@H]2NC(C3=CC=C(NCCCCCCNC4=CC5=C(C(N(C6C(NC(CC6)=O)=O)C5=O)=O)C=C4)C=C3)=O)=C1	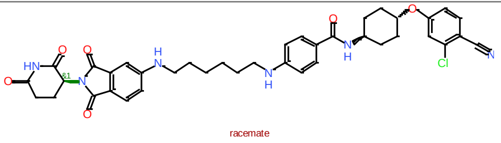

**c60**	15	ClC1=C(C#N)C=CC(O[C@@H](CC2)CC[C@H]2NC(C3=CC=C(NCCCCCCCNC4=CC5=C(C(N(C6C(NC(CC6)=O)=O)C5=O)=O)C=C4)C=C3)=O)=C1	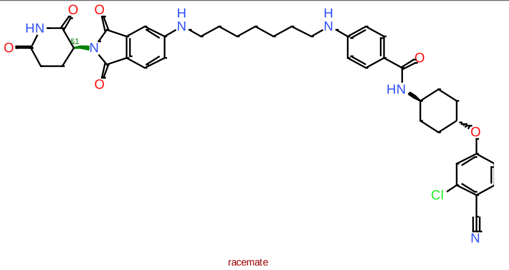
**c61**	16	ClC1=C(C#N)C=CC(O[C@@H](CC2)CC[C@H]2NC(C3=CC=C(NCCCCCCCCNC4=CC5=C(C(N(C6C(NC(CC6)=O)=O)C5=O)=O)C=C4)C=C3)=O)=C1	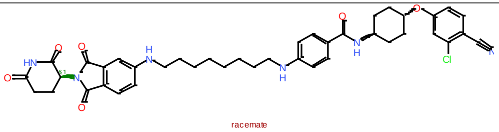
**c62**	17	ClC1=C(C#N)C=CC(O[C@@H](CC2)CC[C@H]2NC(C3=CC=C(NCCCCCCCCCNC4=CC5=C(C(N(C6C(NC(CC6)=O)=O)C5=O)=O)C=C4)C=C3)=O)=C1	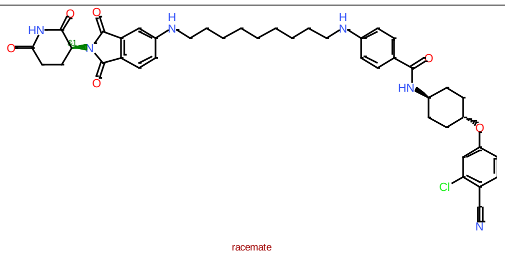
**c63**	18	ClC1=C(C#N)C=CC(O[C@@H](CC2)CC[C@H]2NC(C3=CC=C(NCCCCCCCCCNC4=CC5=C(C(N(C6C(NC(CC6)=O)=O)C5=O)=O)C=C4)C=C3)=O)=C1	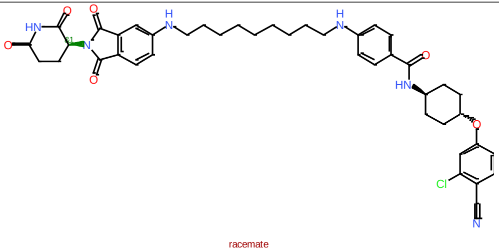
**c64**	19	ClC1=C(C#N)C=CC(O[C@@H](CC2)CC[C@H]2NC(C3=CC=C(NCCCCCCCCCCCNC4=CC5=C(C(N(C6C(NC(CC6)=O)=O)C5=O)=O)C=C4)C=C3)=O)=C1	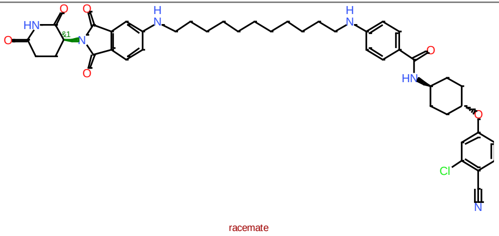
**c65**	20	ClC1=C(C#N)C=CC(O[C@@H](CC2)CC[C@H]2NC(C3=CC=C(N4CCN(CCNC5=CC6=C(C(N(C7C(NC(CC7)=O)=O)C6=O)=O)C=C5)CC4)C=C3)=O)=C1	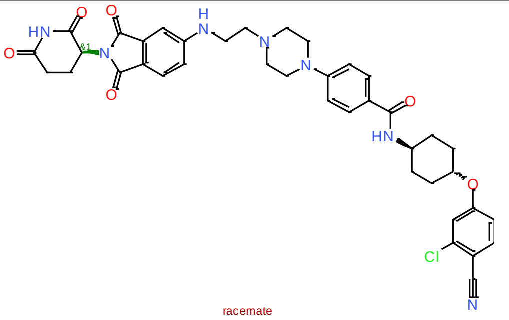

**c66**	21	ClC1=C(C#N)C=CC(O[C@@H](CC2)CC[C@H]2NC(C3=CC=C(N4CCN(CCCNC5=CC6=C(C(N(C7C(NC(CC7)=O)=O)C6=O)=O)C=C5)CC4)C=C3)=O)=C1	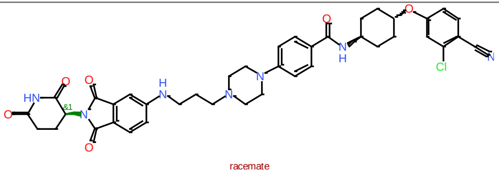
**c67**	22	ClC1=C(C#N)C=CC(O[C@@H](CC2)CC[C@H]2NC(C3=CC=C(N4CCN(CCCCNC5=CC6=C(C(N(C7C(NC(CC7)=O)=O)C6=O)=O)C=C5)CC4)C=C3)=O)=C1	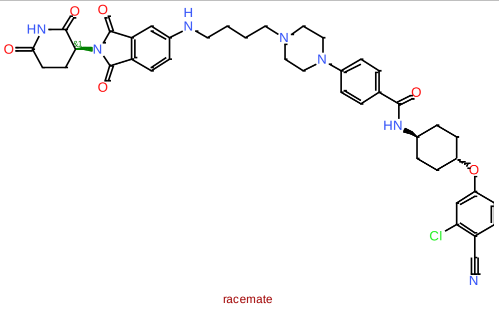
**c68**	23	ClC1=C(C#N)C=CC(O[C@@H](CC2)CC[C@H]2NC(C3=CC=C(N4CCN(CCCCCNC5=CC6=C(C(N(C7C(NC(CC7)=O)=O)C6=O)=O)C=C5)CC4)C=C3)=O)=C1	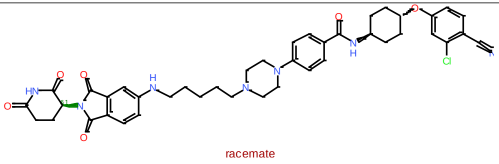
**c69**	24	ClC1=C(C#N)C=CC(O[C@@H](CC2)CC[C@H]2NC(C3=CC=C(N4CCN(CC(CC5)CCN5C6=CC7=C(C(N(C8C(NC(CC8)=O)=O)C7=O)=O)C=C6)CC4)C=C3)=O)=C1	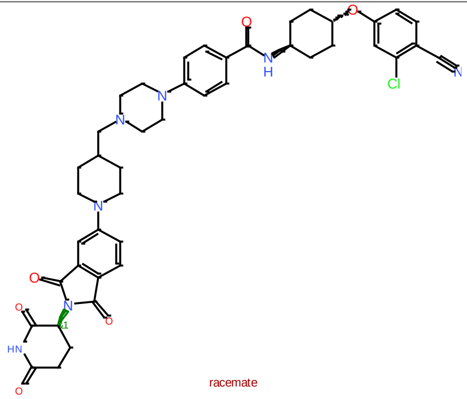
**c70**	25	ClC1=C(C#N)C=CC(O[C@@H](CC2)CC[C@H]2NC(C3=CC=C(N4CCN(C(CC5)CCN5C6=CC7=C(C(N(C8C(NC(CC8)=O)=O)C7=O)=O)C=C6)CC4)C=C3)=O)=C1	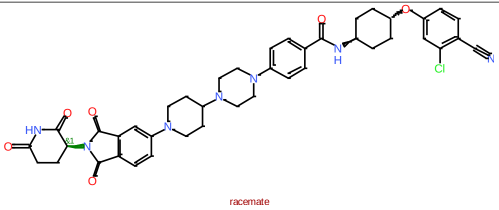
**c71**	26	ClC1=C(C#N)C=CC(O[C@@H](CC2)CC[C@H]2NC(C3=CC=C(N4CCC(N(CC5)CCN5C6=CC7=C(C(N(C8C(NC(CC8)=O)=O)C7=O)=O)C=C6)CC4)C=C3)=O)=C1	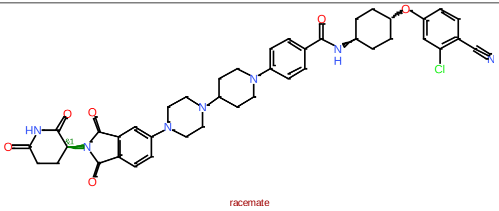

**c72**	27	ClC1=C(C#N)C=CC(O[C@@H](CC2)CC[C@H]2NC(C3=CC=C(N4CCN(CC5CN(C5)C6=CC7=C(C(N(C8C(NC(CC8)=O)=O)C7=O)=O)C=C6)CC4)C=C3)=O)=C1	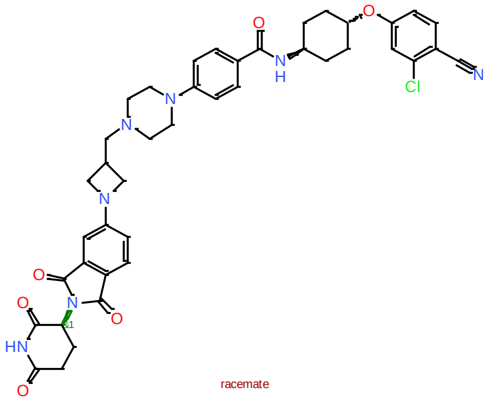
**c73**	28	ClC1=C(C#N)C=CC(O[C@@H](CC2)CC[C@H]2NC(C3=CC=C(N4CCN(C5CN(C5)C6=CC7=C(C(N(C8C(NC(CC8)=O)=O)C7=O)=O)C=C6)CC4)C=C3)=O)=C1	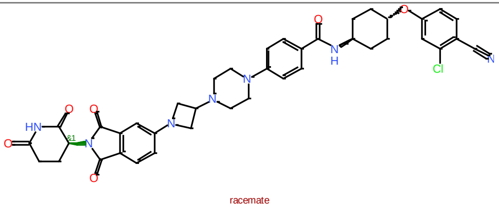
**c74**	29	ClC1=C(C#N)C=CC(O[C@@H](CC2)C[C@H]2NC(C3=CC=C(N4CCC(N(CC5)CCN5C6=CC7=C(C(N(C8C(NC(CC8)=O)=O)C7=O)=O)C=C6)CC4)C=C3)=O)=C1	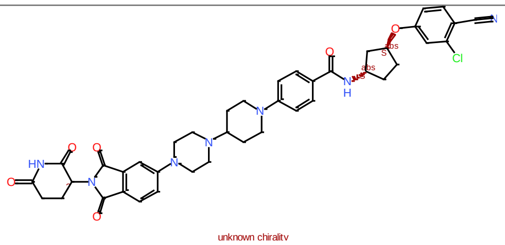
**c75**	30	ClC1=C(C#N)C=CC(O[C@@H](CC2)CCC[C@H]2NC(C3=CC=C(N4CCC(N(CC5)CCN5C6=CC7=C(C(N(C8C(NC(CC8)=O)=O)C7=O)=O)C=C6)CC4)C=C3)=O)=C1	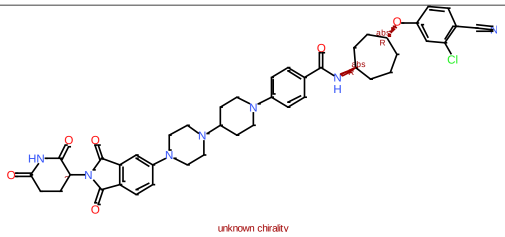
**c76**	31	ClC1=C(C#N)C=CC(O[C@@H](CCC2)CCC[C@@H]2NC(C3=CC=C(N4CCC(N(CC5)CCN5C6=CC7=C(C(N(C8C(NC(CC8)=O)=O)C7=O)=O)C=C6)CC4)C=C3)=O)=C1	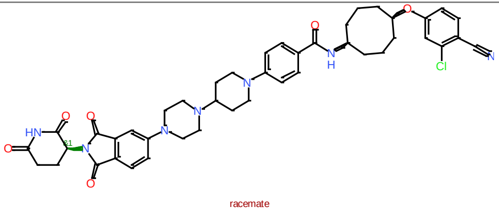

**c77**	32	ClC1=C(C#N)C=CC(OC2CCN(C(C3=CC=C(N4CCC(N(CC5)CCN5C6=CC7=C(C(N(C8C(NC(CC8)=O)=O)C7=O)=O)C=C6)CC4)C=C3)=O)CC2)=C1	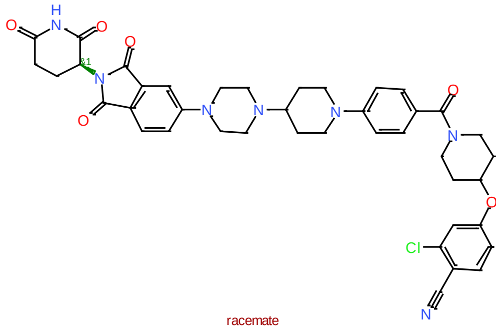
**c78**	33	ClC1=C(C#N)C=CC(OC2CCCN(C(C3=CC=C(N4CCC(N(CC5)CCN5C6=CC7=C(C(N(C8C(NC(CC8)=O)=O)C7=O)=O)C=C6)CC4)C=C3)=O)CC2)=C1	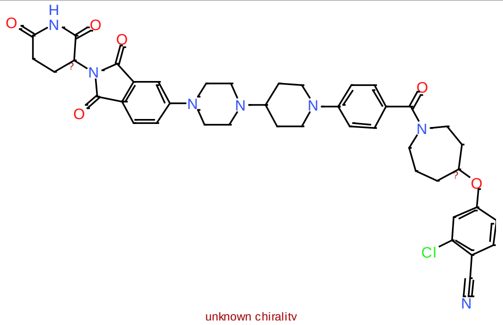
**c79**	34	ClC1=C(C#N)C=CC(N[C@@H](CC2)CC[C@H]2NC(C3=CC=C(N4CCC(N(CC5)CCN5C6=CC7=C(C(N(C8C(NC(CC8)=O)=O)C7=O)=O)C=C6)CC4)C=C3)=O)=C1	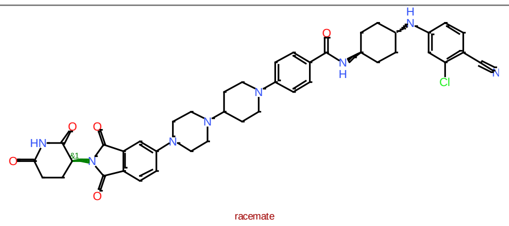
**c80**	35	ClC1=C(C#N)C=CC(N(C)[C@@H](CC2)CC[C@H]2NC(C3=CC=C(N4CCC(N(CC5)CCN5C6=CC7=C(C(N(C8C(NC(CC8)=O)=O)C7=O)=O)C=C6)CC4)C=C3)=O)=C1	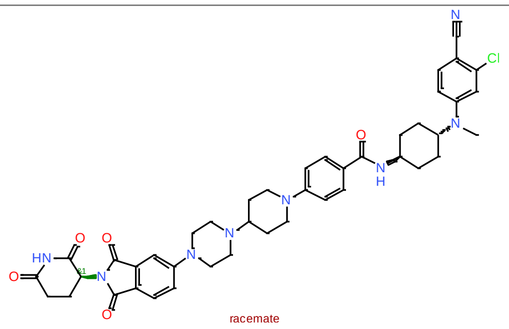
**c81**	36	ClC1=C(C#N)C=CC(N(CC)[C@@H](CC2)CC[C@H]2NC(C3=CC=C(N4CCC(N(CC5)CCN5C6=CC7=C(C(N(C8C(NC(CC8)=O)=O)C7=O)=O)C=C6)CC4)C=C3)=O)=C1	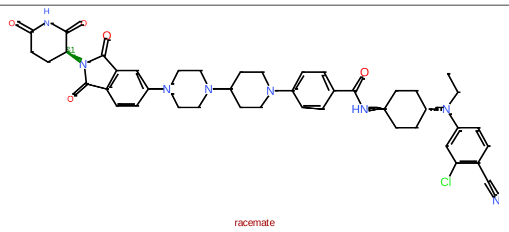
**c82**	37	ClC1=C(C#N)C=CC(N(CCC)[C@@H](CC2)CC[C@H]2NC(C3=CC=C(N4CCC(N(CC5)CCN5C6=CC7=C(C(N(C8	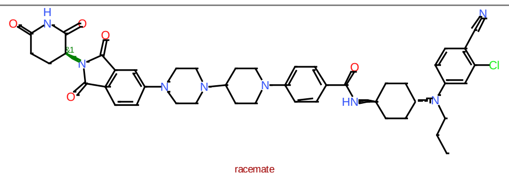

		C(NC(CC8)=O)=O)C7=O)=O)C=C6)CC4)C=C3)=O)=C1	
**c83**	39	ClC1=C(C#N)C=CC(N(C)[C@@H](CC2)CC[C@H]2NC(C3=CC=C(N4CCN(CC(CC5)CCN5C6=CC7=C(C(N(C8C(NC(CC8)=O)=O)C7=O)=O)C=C6)CC4)C=C3)=O)=C1	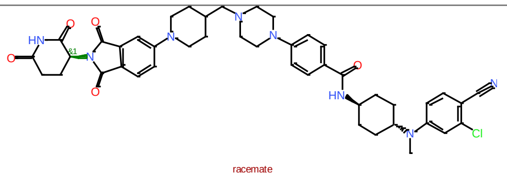
**c84**	40	ClC1=C(C#N)C=CC(N(C)[C@@H](CC2)CC[C@H]2NC(C3=CC=C(N4CCC(CN(CC5)CCN5C6=CC7=C(C(N(C8C(NC(CC8)=O)=O)C7=O)=O)C=C6)CC4)C=C3)=O)=C1	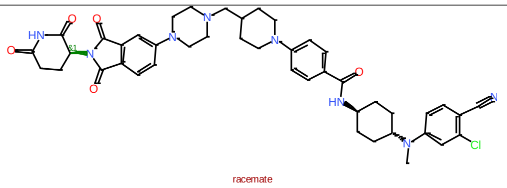
**c85**	41	ClC1=C(C#N)C=CC(N(C)[C@@H](CC2)CC[C@H]2NC(C3=CC=C(N4CCN(C(CC5)CCN5C6=CC7=C(C(N(C8C(NC(CC8)=O)=O)C7=O)=O)C=C6)CC4)C=C3)=O)=C1	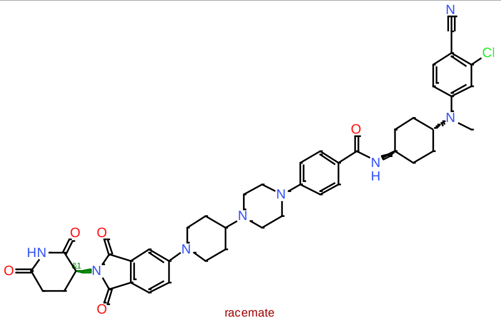
**c86**	42	ClC1=C(C#N)C=CC(N(C)[C@@H](CC2)CC[C@H]2NC(C3=CC=C(N4CCN(CC5CN(C5)C6=CC7=C(C(N(C8C(NC(CC8)=O)=O)C7=O)=O)C=C6)CC4)C=C3)=O)=C1	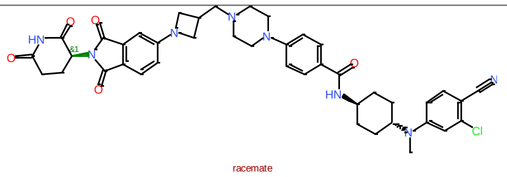
**c87**	43	ClC1=C(C#N)C=CC(N(C)[C@@H](CC2)CC[C@H]2NC(C3=CC=C(N4CCN(C5CN(C5)C6=CC7=C(C(N(C8C(NC(CC8)=O)=O)C7=O)=O)C=C6)CC4)C=C3)=O)=C1	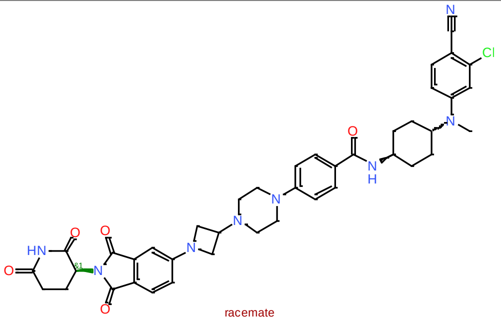
**c88**	44	ClC1=C(C#N)C=CC(N(C)[C@@H](CC2)CC[C@H]2NC(C3=CC=C(N4CC(CN(CC5)CCN5C6=CC7=C(C(N(C8C(NC(CC8)=O)=O)C7=O)=O)C=C6)C4)C=C3)=O)=C1	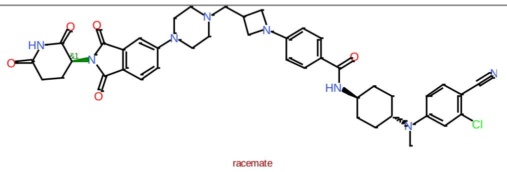

**c89**	45	ClC1=C(C#N)C=CC(N(C)[C@@H](CC2)CC[C@H]2NC(C3=CC=C(N4CC(N(CC5)CCN5C6=CC7=C(C(N(C8C(NC(CC8)=O)=O)C7=O)=O)C=C6)C4)C=C3)=O)=C1	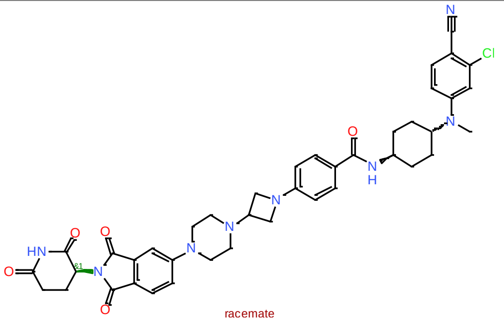 racemate
**c90**	46	O=C(C(O)(C)CS(C1=CC=C(N2CCN(C(CC3)CCN3C4=CC5=C(C(N(C6C(NC(CC6)=O)=O)C5=O)=O)C=C4)CC2)C=C1)(=O)=O)NC7=CC(C(F)(F)F)=C(C#N)C=C7	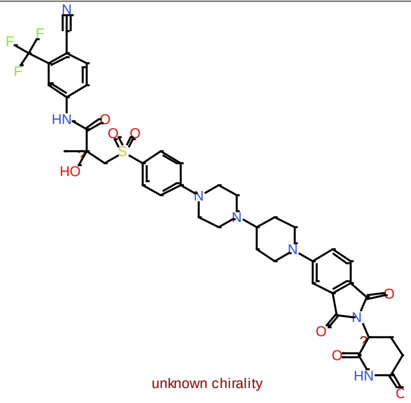 unknown chirality
**c91**	47	O=C1N(C2C(NC(CC2)=O)=O)C(C3=C1C=C(N4CCC(CC4)N5CCN(C(C(C(F)=C6)=CC=C6N7C(C)(C)C(N(C7=S)C8=CC(C(F)(F)F)=C(C#N)C=C8)=O)=O)CC5)C=C3)=O	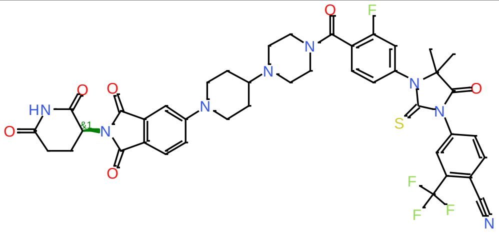 racemate
**c92**	48	O=C1N(C2C(NC(CC2)=O)=O)C(C3=C1C=C(N4CCC(CC4)N5CCN(C(C(C(F)=C6)=CC=C6N7C8(CCC8)C(N(C7=S)C9=CC(C(F)(F)F)=C(C#N)N=C9)=O)=O)CC5)C=C3)=O	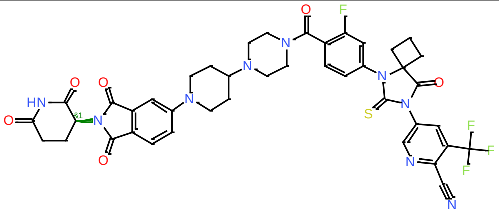 racemate

Table S2: Activity and classification as active or inactive (threshold set at 75% of degradation set); activity reported at both concentrations.

Our notation	Activity (0.1uM)	Activity 75% (0.1uM)	Activity (1uM)	Activity 75% (1uM)
**c01**	95	active	99	active
**c02**	95	active	99	active
**c03**	76	active	95	active
**c04**	95	active	99	active
**c05**	95	active	99	active
**c06**	72	inactive	99	active
**c07**	11	inactive	17	inactive
**c08**	68	inactive	86	active
**c09**	67	inactive	68	inactive
**c10**	95	active	99	active
**c11**	95	active	99	active
**c12**	95	active	99	active
**c13**	95	active	99	active
**c14**	95	active	99	active
**c15**	95	active	99	active
**c16**	95	active	99	active
**c17**	95	active	99	active
**c18**	95	active	99	active
**c19**	95	active	99	active
**c20**	95	active	99	active
**c21**	5	inactive	5	inactive
**c22**	26	inactive	35	inactive
**c23**	15	inactive	23	inactive
**c24**	16	inactive	20	inactive
**c25**	11	inactive	25	inactive
**c26**	54	inactive	84	active
**c27**	29	inactive	65	inactive
**c28**	66	inactive	87	active
**c29**	48	inactive	88	active
**c30**	48	inactive	86	active
**c31**	30	inactive	69	inactive
**c32**	32	inactive	35	inactive
**c33**	63	inactive	96	active
**c34**	50	inactive	80	active
**c35**	61	inactive	92	active
**c36**	48	inactive	89	active
**c37**	51	inactive	78	active
**c38**	3	inactive	31	inactive
**c39**	0	inactive	12	inactive
**c40**	50	inactive	87	active
**c41**	75	active	94	active
**c42**	72	inactive	93	active
**c43**	81	active	97	active
**c44**	80	active	95	active

**c45**	18	inactive	34	inactive
**c46**	98	active	99	active
**c47**	65	inactive	93	active
**c48**	99	active	100	active
**c49**	99	active	100	active
**c50**	77	active	99	active
**c51**	5	inactive	5	inactive
**c52**	5	inactive	5	inactive
**c53**	91	active	96	active
**c54**	7	inactive	20	inactive
**c55**	2	inactive	25	inactive
**c56**	61	inactive	48	inactive
**c57**	55	inactive	38	inactive
**c58**	82	active	79	active
**c59**	86	active	82	active
**c60**	62	inactive	38	inactive
**c61**	65	inactive	46	inactive
**c62**	58	inactive	53	inactive
**c63**	1	inactive	22	inactive
**c64**	39	inactive	35	inactive
**c65**	39	inactive	21	inactive
**c66**	80	active	59	inactive
**c67**	80	active	62	inactive
**c68**	51	inactive	34	inactive
**c69**	76	active	75	active
**c70**	60	inactive	43	inactive
**c71**	85	active	75	active
**c72**	85	active	86	active
**c73**	15	inactive	0	inactive
**c74**	23	inactive	18	inactive
**c75**	34	inactive	43	inactive
**c76**	51	inactive	47	inactive
**c77**	0	inactive	4	inactive
**c78**	9	inactive	29	inactive
**c79**	52	inactive	43	inactive
**c80**	93	active	93	active
**c81**	97	active	95	active
**c82**	67	inactive	52	inactive
**c83**	87	active	76	active
**c84**	93	active	90	active
**c85**	99	active	95	active
**c86**	86	active	73	inactive
**c87**	89	active	78	active
**c88**	95	active	94	active
**c89**	88	active	75	active
**c90**	0	inactive	27	inactive
**c91**	18	inactive	31	inactive
**c92**	0	inactive	5	inactive

Table S3. Entire degrader scaffold Matched Molecular Pair applied to Bemis-Murcko framework.

Class	Number of compounds	Framework structural features
**A**	8	c10, c16-c20 share the same scaffold, differences are related to the pendant group linked to the E3 ligand phenyl moiety; c15 and c21 present a different scaffold.
**B**	11	All compounds are characterized by the same warhead and E3 ligand scaffolds.
**C**	10	c01-c06 share the same scaffold, differences are related to the E3 ligand pendant group linked to the phenyl moiety; c07, c08, c09, c46 present a different scaffold.
**D**	5	c48, c49, c52 share the same scaffold, differences are related to the E3 ligand pendant group linked to the cyclopropyl ring; c50, c51 present a different scaffold.
**E**	9	All compounds are characterized by the same warhead and E3 ligand scaffold, differing only for the linker length.
**F**	4	All compounds are characterized by the same warhead and E3 ligand scaffold, differing only for the linker length.
**G**	5	Compounds differing only for the size and composition of the ring included in the linker and the presence or absence of a methylene bridge between the ring systems of this moiety.
**H**	5	Compounds differing only for the size and composition of the central ring included in the warhead moiety.
**I**	11	Compounds differing for the length and composition of the alkyl moiety included in the linker.
**L**	7	Compounds differing for the linker composition.
**M**	4	Compounds differing for the size and composition of the rings included in the linker moiety.
**N**	13	No structural features shared (outliers)

Table S4. Warhead skeleton Matched Molecular Pair applied to Bemis-Murcko framework.

Class	Number of compounds	Framework structural features
**AR-a**	30	Three ring system, two six membered and one four membered, which were connected by alkyl moieties
**AR-b**	34	Three ring system, two six membered and one which include from five up to eight atoms, connected by alkyl moieties
**AR-c**	25	Three ring systems, two six membered and one five membered, directly connected
**AR-d**	3	No structural features shared (outliers)

Table S5. E3 ligand skeleton Matched Molecular Pair applied to Bemis-Murcko framework.

Class	Number of compounds	Structural features
**VHL-a**	23	Core structure formed by a five-membered ring directly connected to a six-membered ring system, where the last ring system is connected through an alkyl moiety to a different five-membered ring group.
**VHL-b**	30	Core structure formed by a six-membered ring linked through an alkyl moiety to a five-membered ring
**CRBN**	39	Thalidomide substucture

Table S6. Linker scaffold Matched Molecular Pair applied to Bemis-Murcko framework.

Class	Number of compounds	Structural features
**LA (long alkyl)**	18	More than or exactly five methylene groups in the chain
**SA (short alkyl)**	4	Less than five methylene groups in the chain
**P (pegylated)**	1	Poly (ethylene glycole)-based chain
**A (aromatic)**	9	Aromatic ring inclusion
**Ca (cyclic-a)**	33	Two six-membered ring directly connected
**Cb (cyclic-b)**	7	A six-membered ring directly connected to a four-membered ring
**Cc (cyclic-c)**	6	Two ring systems connected through a methylene bridge
**Cd (cyclic-d)**	14	A single six-membered ring

Table S7. Degradation Cliffs table reporting the degraders involved, the DCs compounds name, delta activity at both concentrations and the similarity value.

Degraders involved	Moiety involved	DCs	Delta Activity (0,1uM)	Delta Activity (1uM)	Similarity
CRBN-degraders	Warhead	C71 - C77	84	71	0.951
		C87 - C73	74	77	0.944
CRBN-degraders	Warhead + linker	C89 - C73	73	74	0.926
**CRBN-degraders**	Linker	C72 - C73	70	85	0.966
		C71 - C73	70	74	0.961
**VHL-degraders**	E3 ligand	C48 - C51	94	95	0.995
		C48 - C52	94	95	0.986
		C49 - C51	94	95	0.968
		C49 - C52	94	95	0.956
		C46 - C52	93	94	0.931
		C10 - C21	90	94	0.952
		C50 - C07	66	82	0.923
		C50 - C51	72	94	0.970
		C50 - C52	72	94	0.961
		C42 - C39	71	81	0.920

Table S8. Performance metrics for classification models with 75% degradation activity as active/non-active threshold for 0.1uM activity for VHL dataset.

VHL models	Sensitivity (TPR)	Specificity (TNR)	MCC	ROC Area
**RF (training)**	0.737	0.913	0.666	0.863
**RF (test)**	0.800	0.833	0.633	0.867
**RT (training)**	0.737	0.870	0.615	0.819
**RT (test)**	0.800	0.833	0.633	0.817
**NB (training)**	0.737	0.783	0.519	0.763
**NB (test)**	0.600	0.833	0.449	0.733
**KNN (training)**	0.789	0.783	0.570	0.791
**KNN (test)**	0.600	0.833	0.449	0.917
**SVM (training)**	0.684	0.867	0.551	0.742
**SVM (test)**	0.778	0.800	0.578	0.800

Each model appears twice in the table, the first time showing the performance metrics of the training set during the 10-fold cross-validation, and the second the values obtained during the external validation on the test set. KNN model was implemented with k=5. RF: Random Forest; RT: Random Tree; NB: Naïve Bayes; KNN: K-nearest neighbors; SVM: Support Vector Machine (linear kernel)

Table S9. Performance metrics for classification models with 75% degradation activity as active/non-active threshold for 0.1uM activity for CRBN dataset.

CRBN models	Sensitivity (TPR)	Specificity (TNR)	MCC	ROC Area
**RF (training)**	0.556	0.615	0.169	0.615
**RF (test)**	0.800	1.000	0.775	0.967
**RT (training)**	0.444	0.692	0.139	0.568
**RT (test)**	1.000	0.667	0.745	0.833
**NB (training)**	0.556	0.769	0.325	0.615
**NB (test)**	0.800	1.000	0.775	0.833
**KNN (training)**	0.444	0.692	0.139	0.515
**KNN (test)**	0.800	1.000	0.775	0.933
**SVM (training)**	0.643	0.714	0.337	0.667
**SVM (test)**	0.300	0.750	0.055	0.362

Each model appears twice in the table, the first time showing the performance metrics of the training set during the 10-fold cross-validation, and the second the values obtained during the external validation on the test set. KNN model was implemented with k=5. RF: Random Forest; RT: Random Tree; NB: Naïve Bayes; KNN: K-nearest neighbors; SVM: Support Vector Machine (linear kernel)

Table S10. Performance metrics for classification models with 75% degradation activity as active/non-active threshold for 0.1uM activity for the entire dataset.

Entire dataset models	Sensitivity (TPR)	Specificity (TNR)	MCC	ROC Area
**RF (training)**	0.781	0.690	0.468	0.770
**RF (test)**	0.750	0.700	0.447	0.813
**RT (training)**	0.750	0.643	0.390	0.709
**RT (test)**	0.625	0.600	0.224	0.644
**NB (training)**	0.563	0.571	0.133	0.620
**NB (test)**	0.875	0.600	0.484	0.763
**KNN (training)**	0.781	0.619	0.399	0.805
**KNN (test)**	0.875	0.600	0.484	0.750
**SVM (training)**	0.714	0.857	0.539	0.737
**SVM (test)**	0.588	0.800	0.399	0.631

Each model appears twice in the table, the first time showing the performance metrics of the training set during the 10-fold cross-validation, and the second the values obtained during the external validation on the test set. KNN model was implemented with k=5. RF: Random Forest; RT: Random Tree; NB: Naïve Bayes; KNN: K-nearest neighbors; SVM: Support Vector Machine (linear kernel)

Table S11. Performance metrics for classification models with 75% degradation activity as active/non-active threshold for 1uM activity for VHL dataset.

VHL models	Sensitivity (TPR)	Specificity (TNR)	MCC	ROC Area
**RF (training)**	0.828	0.538	0.375	0.775
**RF (test)**	1.000	0.000	-	0.833
**RT (training)**	0.690	0.615	0.287	0.694
**RT (test)**	1.000	0.500	0.671	0.722
**NB (training)**	0.897	0.385	0.331	0.696
**NB (test)**	1.000	0.500	0.671	0.944
**KNN (training)**	0.897	0.385	0.331	0.635
**KNN (test)**	1.000	0.000	-	0.833
**SVM (training)**	0.870	0.727	0.597	0.925
**SVM (test)**	0.857	0.400	0.278	0.850

Each model appears twice in the table, the first time showing the performance metrics of the training set during the 10-fold cross-validation, and the second the values obtained during the external validation on the test set. KNN model was implemented with k=5. RF: Random Forest; RT: Random Tree; NB: Naïve Bayes; KNN: K-nearest neighbors; SVM: Support Vector Machine (linear kernel)

Table S12. Performance metrics for classification models with 75% degradation activity as active/non-active threshold for 1uM activity for CRBN dataset.

CRBN models	Sensitivity (TPR)	Specificity (TNR)	MCC	ROC Area
**RF (training)**	0.714	0.400	0.114	0.581
**RF (test)**	0.800	1.000	0.775	0.967
**RT (training)**	0.667	0.400	0.065	0.490
**RT (test)**	1.000	0.667	0.745	0.967
**NB (training)**	0.571	0.700	0.254	0.562
**NB (test)**	0.800	1.000	0.775	0.833
**KNN (training)**	0.667	0.500	0.160	0.521
**KNN (test)**	0.800	1.000	0.775	0.833
**SVM (training)**	0.643	0.857	0.472	0.805
**SVM (test)**	0.250	0.900	0.200	0.711

Each model appears twice in the table, the first time showing the performance metrics of the training set during the 10-fold cross-validation, and the second the values obtained during the external validation on the test set. KNN model was implemented with k=5. RF: Random Forest; RT: Random Tree; NB: Naïve Bayes; KNN: K-nearest neighbors; SVM: Support Vector Machine (linear kernel)

Table S13. Performance metrics for classification models with 75% degradation activity as active/non-active threshold for 1uM activity for the entire dataset.

Entire dataset models	Sensitivity (TPR)	Specificity (TNR)	MCC	ROC Area
**RF (training)**	0.769	0.629	0.403	0.736
**RF (test)**	0.677	0.833	0.471	0.875
**RT (training)**	0.744	0.571	0.320	0.684
**RT (test)**	0.750	0.667	0.403	0.771
**NB (training)**	0.718	0.686	0.404	0.671
**NB (test)**	0.667	0.500	0.161	0.569
**KNN (training)**	0.923	0.486	0.460	0.733
**KNN (test)**	0.917	0.833	0.750	0.861
**SVM (training)**	0.800	0.750	0.540	0.800
**SVM (test)**	0.500	0.800	0.304	0.717

Each model appears twice in the table, the first time showing the performance metrics of the training set during the 10-fold cross-validation, and the second the values obtained during the external validation on the test set. KNN model was implemented with k=5. RF: Random Forest; RT: Random Tree; NB: Naïve Bayes; KNN: K-nearest neighbors; SVM: Support Vector Machine (linear kernel)

Table S14. Y-randomization results of 0.1uM concentrations activity models on the training set.

Model	Sensitivity (TPR)	Specificity (TNR)	MCC	ROC Area
**VHL models**				
RF (training)	0.476	0.450	-0.095	0.458
RT (training)	0.524	0.450	-0.048	0.442
NB (training)	0.429	0.500	-0.048	0.390
KNN (training)	0.429	0.450	-0.143	0.338
SVM (training)	0.565	0.636	0.189	0.375
**CRBN models**				
RF (training)	0.706	0.286	-0.009	0.517
RT (training)	0.529	0.286	-0.189	0.464
NB (training)	0.529	0.571	0.100	0.475
KNN (training)	0.706	0.071	-0.281	0.454
SVM (training)	0.545	0.500	0.045	0.314
**Entire dataset models**				
RF (training)	0.342	0.472	-0.187	0.391
RT (training)	0.368	0.417	-0.215	0.393
NB (training)	0.447	0.667	0.117	0.515
KNN (training)	0.395	0.306	-0.301	0.337
SVM (training)	0.650	0.571	0.213	0.317

RF: Random Forest; RT: Random Tree; NB: Naïve Bayes; KNN: K-nearest neighbors; SVM: Support Vector Machine (linear kernel)

Table S15. 3-descriptors models (TPSA, nHAcc, MW) implemented using Random Forest algorithm performed on 0.1uM activity data**.**

**Model**		**Sensitivity (TPR)**	**Specificity (TNR)**	**MCC**	**Roc Area**
**VHL**	RF training	0.737	0.870	0.615	0.839
	RF test	0.800	0.833	0.633	0.767
**CRBN**	RF training	0.500	0.538	0.038	0.603
	RF test	0.800	1.000	0.775	0.967
**Entire dataset**	RF training	0.594	0.619	0.211	0.711
	RF test	0.875	0.800	0.671	0.888

Each model appears twice in the table, the first time showing the performance metrics of the training set during the 10-fold cross-validation, and the second the values obtained during the external validation on the test set. RF: Random Forest

Table S16. Crystallographic structures employed in ternary complex modelling.

PDB ID	Description
**1Z95**	AR W741L in complex with Bicalutamide
**5NVV**	VHL in complex with VH032 derivative
**5NVX**	VHL in complex with VH101
**5LLI**	BHL in complex with VH298
**4TZ4**	CRBN in complex with Lenalidomide
